# Trans- and cis-acting effects of *Firre* on epigenetic features of the inactive X chromosome

**DOI:** 10.1038/s41467-020-19879-3

**Published:** 2020-11-27

**Authors:** He Fang, Giancarlo Bonora, Jordan P. Lewandowski, Jitendra Thakur, Galina N. Filippova, Steven Henikoff, Jay Shendure, Zhijun Duan, John L. Rinn, Xinxian Deng, William S. Noble, Christine M. Disteche

**Affiliations:** 1 Department of Laboratory Medicine and Pathology, University of Washington, Seattle, WA USA; 2 Department of Genome Sciences, University of Washington, Seattle, WA USA; 3 Department of Stem Cell and Regenerative Biology, Harvard University, Boston, MA USA; 4 Fred Hutchinson Cancer Research Center, Seattle, WA USA; 5 Institute for Stem Cell and Regenerative Medicine, University of Washington, Seattle, WA USA; 6 Division of Hematology, Department of Medicine, University of Washington, Seattle, WA USA; 7 Department of Biochemistry, University of Colorado at Boulder, Boulder, CO USA; 8 Paul G. Allen School of Computer Science and Engineering, University of Washington, Seattle, WA USA; 9 Department of Medicine, University of Washington, Seattle, WA USA

**Keywords:** Nuclear organization, Epigenetics, Chromatin structure, Long non-coding RNAs, Nuclear organization

## Abstract

*Firre* encodes a lncRNA involved in nuclear organization. Here, we show that *Firre* RNA expressed from the active X chromosome maintains histone H3K27me3 enrichment on the inactive X chromosome (Xi) in somatic cells. This trans-acting effect involves SUZ12, reflecting interactions between *Firre* RNA and components of the Polycomb repressive complexes. Without *Firre* RNA, H3K27me3 decreases on the Xi and the Xi-perinucleolar location is disrupted, possibly due to decreased CTCF binding on the Xi. We also observe widespread gene dysregulation, but not on the Xi. These effects are measurably rescued by ectopic expression of mouse or human *Firre*/*FIRRE* transgenes, supporting conserved trans-acting roles. We also find that the compact 3D structure of the Xi partly depends on the *Firre* locus and its RNA. In common lymphoid progenitors and T-cells *Firre* exerts a cis-acting effect on maintenance of H3K27me3 in a 26 Mb region around the locus, demonstrating cell type-specific trans- and cis-acting roles of this lncRNA.

## Abstract (web summary)

*Firre* encodes a lncRNA involved in nuclear organization in mammals. Here, the authors find that allelic deletion of *Firre* on the active X chromosome (Xa) results in dose-dependent loss of histone H3K27me3 on the inactive X chromosome (Xi), along with other trans-acting effects, including disruption of the perinuclear location and minor dysregulation of gene expression.

## Introduction

X chromosome inactivation (XCI) is initiated by the long noncoding RNA (lncRNA) *Xist*, which becomes highly expressed on one allele, and coats the future inactive X chromosome (Xi) in cis^1–5^. Specific proteins that include components of the Polycomb repressive complexes PRC1 and PRC2 are recruited by *Xist* RNA to mediate serial layers of epigenetic modifications, resulting in gene silencing and heterochromatin formation^2,6,7^. Epigenetic hallmarks of the Xi include multiple repressive histone modifications such as ubiquitination of histone H2A at lysine 119 (H2AK119ubi), tri-methylation of histone H3 at lysine 27 (H3K27me3), and enrichment in the histone variant macroH2A1^8^. Additional layers of control ensure stability of the silent state of the Xi, including DNA methylation of promoter-containing CpG islands, a shift to late replication, and spatial reorganization of the Xi within the nucleus^9,10^.

The Xi appears as the heteropycnotic Barr body usually located close to either the nuclear lamina or the periphery of the nucleolus^11–15^. These two locations are preferred sites of heterochromatin, not only for the Xi but also for other repressed regions of the genome, suggesting that their proximity helps maintain silent chromatin^11,16^. In particular, the perinucleolar space has a primary function in replication and maintenance of repressive chromatin state^17,18^. The factors and mechanisms that facilitate association of heterochromatic regions including the Xi to specific nuclear compartments such as the lamina or the nucleolus remain elusive. *Xist* RNA interaction with the lamin B receptor (LBR) has been proposed as a critical factor that recruits the Xi to the lamina and facilitates silencing^19^. Our previous studies suggest that perinucleolar positioning of the Xi may be facilitated by the lncRNA *Firre*^20^.

The *Firre* locus comprises conserved tandem repeats that bind CTCF specifically on the Xi but not on the Xa (active X chromosome)^20–22^. Despite sequence divergence between species, the conserved nature of the repeat locus suggests important roles in mammals^21^. *Firre* RNA is usually confined to the nucleus where it interacts with the nuclear matrix protein hnRNPU^23,24^. Multiple transcript isoforms including circular RNAs, further complicate an understanding of the roles of *Firre* in different cell types^25^. On the Xi the *Firre* locus contacts the *Dxz4* locus that also binds CTCF only on the Xi^26–28^. *Dxz4* is necessary for the formation of the bipartite structure of the Xi^27,29–31^. The *Firre* locus also interacts with several autosomal regions, consistent with a widespread role in nuclear architecture^23,32^. A *Firre* knockout (KO) mouse model is viable, but results in cell-type-specific defects in hematopoiesis that impact common lymphoid progenitors (CLPs)^32,33^. Importantly, these defects are rescued by ectopic expression of *Firre* from an autosomal location, thus defining a trans-acting role for *Firre*^32,33^. KO mice show organ-specific dysregulation of autosomal genes, consistent with physiological defects in distinct phases of hematopoiesis^32^.

Here, we investigate the role of *Firre* in maintenance of heterochromatin, gene expression, and 3D structure of the Xi by engineering allele-specific deletions of the *Firre* locus and by *Firre* knockdown (KD) in mouse cell lines and tissues. Depletion of *Firre* RNA reveals important roles in H3K27me3 enrichment on the Xi and in location of the Xi within the nucleus as shown by immunostaining, ChIP-seq, and CUT&RUN. Gene expression is disrupted, as is the 3D structure of the Xi as shown by RNA-seq, ATAC-seq, and Hi-C. Our results are supported by rescue experiments using cDNA transgenes. We demonstrate both trans- and cis-acting roles of *Firre* RNA and its locus, with evidence of cell-type-specific effects in cell lines and in vivo.

## Results

### *Firre* and *CrossFirre* are transcribed from the Xa

**Fig. 1:**
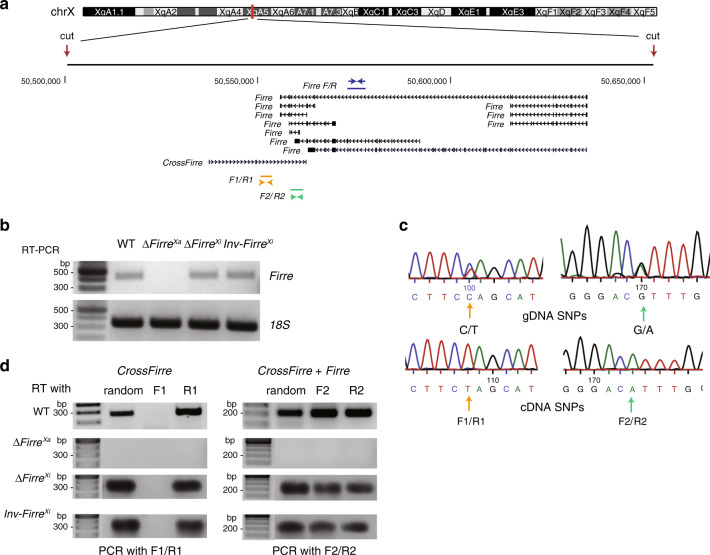
*Firre* and *CrossFirre* are expressed from the Xa. **a** Genomic location of *Firre* and *CrossFirre* (*Gm35612*) on the mouse X chromosome (UCSC mm10 build 38 browser tracks). Note that *Firre* has multiple alternative transcripts. The locations of the CRISPR guide RNAs used to edit the locus (cut), and of the RT-PCR primer pairs to specifically detect *Firre* expression (F/R), strand-specific expression of *CrossFirre* (F1/R1), or strand-specific expression in a region of overlap between *Firre* and *CrossFirre* (F2/R2) are indicated. **b** RT-PCR analysis using the F/R primer pair detects *Firre* expression in WT, Δ*Firre*^Xi^, and Inv*Firre*^Xi^, but not in Δ*Firre*^Xa^. *Firre* expression was measured in *n* = 3 biologically independent samples per cell type. **c** Sanger sequencing analyses of a *CrossFirre* region (F1/R1) and of a region of overlap between *Firre* and *CrossFirre* (F2/R2) confirm heterozygosity of SNPs (BL6 on the Xi and *spretus* on the Xa) in each region assayed. Genomic DNA (gDNA) shows heterozygosity at the SNPs, while cDNA only shows expression from the *spretus* SNP (Xa). **d** Strand-specific analysis of *CrossFirre* and *Firre* was done using reverse transcription using either random primers, or F1, R1, F2, R2 primers, followed by PCR using F1/R1 or F2/R2 primer pairs. *Firre* and *CrossFirre* expression was measured in *n* = 3 biologically independent samples per cell type. Source data are provided as a Source Data file.

Allele-specific CRISPR/Cas9 editing of the *Firre* region was done in Patski cells, in which skewed XCI and species-specific SNPs allowed us to design guides to target the Xi from BL6 or the Xa from *Mus spretus* (*spretus*) (Supplementary Data 1). We isolated single-cell clones with either a ~160 kb deletion of *Firre* on the Xa (Δ*Firre*^Xa^), a ~160 kb deletion of *Firre* on the Xi (Δ*Firre*^Xi^), or a ~160 kb inversion of *Firre* on the Xi (Inv*Firre*^Xi^) (Fig. 1a). Deletion of the *Firre* locus on the Xa resulted in undetectable *Firre* expression by RT-PCR, while deletion on the Xi caused no change (Fig. 1a, b, Supplementary Data 2). Allele-specific RNA-seq analysis confirmed the absence of *Firre* transcripts from either Xa or Xi in Δ*Firre*^Xa^, while control loci (*Dxz4*, *Xist*) showed no change (Supplementary Data 3).

Our Δ*Firre*^Xa^ deletion includes the antisense transcript *CrossFirre* that partially overlaps *Firre* (Fig. 1a; Supplementary Fig. 1a)^34^. Note that, in contrast to Δ*Firre*^Xa^, previously constructed *Firre* deletions in mouse ES cells and in a KO mouse do not include all of *CrossFirre*^32,35^. To test *CrossFirre* expression in edited Patski cells, we processed strand-specific RT-PCR using primers (F1/R1) that flank a 231 bp region in the middle of *CrossFirre* with no *Firre* overlap, and primers (F2/R2) that flank a 203 bp region overlapping the 3’ end of *Firre* (Fig. 1a). For the non-overlapped region, forward *CrossFirre* transcripts containing only SNPs from the Xa (*spretus*), were present in WT (wild-type), Δ*Firre*^Xi^, and Inv*Firre*^Xi^, but absent in Δ*Firre*^Xa^ (Fig. 1c, d). For the overlapped region, transcripts from both directions (*CrossFirre* and *Firre*), again containing only SNPs from the Xa (*spretus*), were present in WT, Δ*Firre*^Xi^, and Inv*Firre*^Xi^, but absent in Δ*Firre*^Xa^ (Fig. 1d). We found no evidence of miRNAs in the *Firre/CrossFirre* region using miRNA-seq in WT or Δ*Firre*^Xa^, suggesting that the loci function independently of the small RNA pathway (Supplementary Data 4).

We conclude that *Firre* and *CrossFirre* are both transcribed from the Xa in Patski cells. Note that *Firre* was originally identified as a gene that escapes XCI in human and mouse^20,23,28^. However, our current results and those reported in *Firre* KO mouse ES cells and in KO mice clearly show that *Firre* is predominantly expressed from the Xa in fibroblasts^32,35^.

### *Firre* acts in trans to maintain PRC2 and H3K27me3 on the Xi

**Fig. 2:**
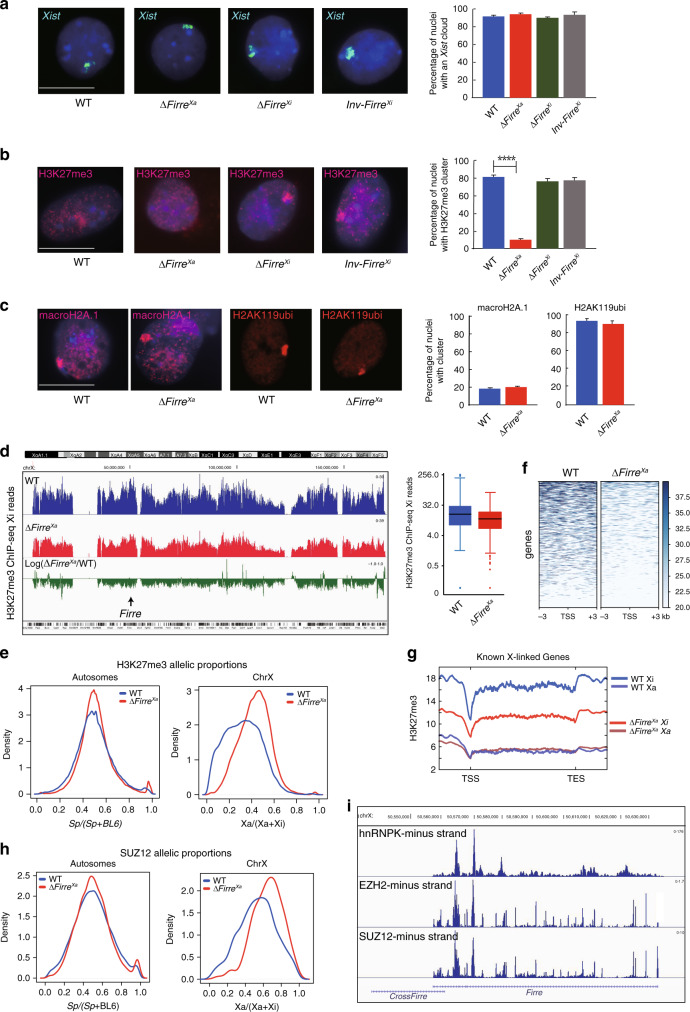
*Firre* RNA acts in trans to maintain H3K27me3 on the Xi. **a**–**c** A total of >300 Patski nuclei were scored per cell type over 3 independent experiments; significance was determined by one-sided Fisher exact test; bar plots are presented as mean values ± SEM; scale bars represent 10 µm. **a** Examples of nuclei after *Xist* RNA-FISH (green) and Hoechst 33342 staining (blue). The bar plot shows no significant differences among cell lines. **b** Examples of nuclei after H3K27me3 immunostaining (red) and Hoechst 33342 staining (blue). The bar plot shows significantly fewer nuclei with a H3K27me3 cluster in Δ*Firre*^Xa^ versus WT (*p* value = 4.63688e-94), but no change in Δ*Firre*^Xi^ nor Inv*Firre*^Xi^. **c** Examples of nuclei after macroH2A.1 or H2AK119ubi (red) immunostaining and Hoechst 33342 staining (blue). The bar plots show no significant differences between cell types. **d** Profiles of H3K27me3 ChIP-seq reads along the Xi in WT (blue), Δ*Firre*^Xa^ (red), and log_2_ ratio Δ*Firre*^Xa^/WT (green). Box plots (log_2_) of H3K27me3 ChIP-seq reads in 100 bp Xi bins show a significantly lower median in Δ*Firre*^Xa^ (red) versus WT (blue) (Wilcoxon test: *p* value = 2.2e-16). The boxes demarcate the interquartile range (IQR) with median; whiskers ±1.5 times the IQR; outliers plotted as points. **e** Density histograms of the distribution of allelic proportions (Xa/(Xa+Xi)) of H3K27me3 peaks show a shift for the X chromosomes due to lower H3K27me3 on the Xi in Δ*Firre*^Xa^ (red) compared to WT (blue) (Wilcoxon test: −log10P = inf). **f** Heatmaps of H3K27me3 ChIP-seq reads 3 kb around transcription start sites (TSS) of genes on the Xi in Δ*Firre*^Xa^ versus WT. **g**. Metagene plots of average H3K27me3 occupancy at X-linked genes ((TSS to termination site (TES), not at scale)) in Δ*Firre*^Xa^ (Xi red Xa pink) versus WT (Xi blue, Xa purple). **h** Density histograms of the distribution of allelic proportions (Xa/(Xa+Xi)) of SUZ12 peaks show a shift for the X chromosomes due to lower SUZ12 on the Xi in Δ*Firre*^Xa^ (red) versus WT (blue) (Wilcoxon test: −log10P = 20.98). **i** Genome tracks demonstrating interactions between hnRNPK, EZH2, and SUZ12 with *Firre* RNA based on RIP-seq data in trophoblast and embryo stem cells^39,40^.

To determine whether any of the allelic alterations constructed, Δ*Firre*^Xa^, Δ*Firre*^Xi^, and Inv*Firre*^Xi^, influences epigenetic marks on the Xi, immunostainings for H3K27me3, H2AK119ubi, and macroH2A.1 were done in combination with *Xist* RNA-FISH to locate the Xi. The majority of nuclei (95 ± 3%) had one *Xist* cloud in all edited cell lines, indicating no disruption of *Xist* RNA coating (Fig. 2a). A strong H3K27me3 immunostaining cluster was observed on the Xi in 83 ± 2% of WT nuclei as expected. In contrast, only 9 ± 2% of nuclei with a H3K27me3 cluster were observed in Δ*Firre*^Xa^, with most nuclei appearing uniformly mottled throughout (Fig. 2b; Table 1). There was no evidence of a complete loss of H3K27me3 over the Xi, which would have appeared as a “hole” with complete absence of immunostaining. H3K27me3 level throughout Δ*Firre*^Xa^ and WT nuclei was measured in regions outside the Xi using ImageJ to quantify fluorescence intensity by normalization to DNA or to histone panH4^36^. No significant difference was detected in regions outside the Xi between Δ*Firre*^Xa^ and WT, but subtle or local changes undetectable by immunostaining cannot be excluded (Supplementary Fig. 2a, b). Δ*Firre*^Xi^ and Inv*Firre*^Xi^ nuclei retained a strong H3K27me3 cluster on the Xi, consistent with retention of *Firre* RNA in these cells (Fig. 2b). Two other histone modifications known to be associated with XCI, H2AK119ubi and macroH2A.1, showed no changes in Δ*Firre*^Xa^ nuclei (Fig. 2c).

**Table 1:** Percentages of nuclei with a H3K27me3 cluster on the Xi.

Cell lines	Genotype	Percent nuclei
Patski cells derived from kidney from an F1 embryo (BL6 *Hprt*^*BM3*^ × *spretus*)	WT	83%
	∆*Firre*^Xa^	9%
	∆*Firre*^Xa+mtransgene^	38%
	∆*Firre*^Xa+htransgene^	21%
	∆*Firre*^Xi^	77%
	Inv*Firre*^Xi^	79%
	*Firre* shRNA KD	33%
	*Firre* siRNA KD	27%
	*Firre* si/shRNA KD	15%
Primary MEFs derived from an F1 embryo (BL6 × *spretus*)	Control	79%
	*Firre* KD	43%
Primary MEFs derived from an F1 embryo with skewed XCI (BL6 *Xist*^*Δ*^ × *spretus*)	Control	80%
	*Firre* KD	45%
Primary MEFs derived from an F1 embryo (BL6 × *castaneus*)	Control	78%
	*Firre* KD	47%
Primary MEFs derived from a *Firre* KO mouse model, with or without induction of *Firre* by doxycycline	*Firre*^+/+^ control	58%
	*Firre*^+/−^ heterozygote	43%
	*Firre*^−/−^ homozygote	41%
	*Firre*^*+/*−^ *tg;rtTA; -Dox*	52%
	*Firre*^*+/*−^ *tg;rtTA; +Dox*	91%
	*Firre*^−*/*−^ *tg;rtTA; -Dox*	49%
	*Firre*^−/−^ tg;rtTA; +Dox	83%
Tissues derived from a *Firre* KO mouse model	*Firre*^+/+^ brain	71%
	*Firre*^−/−^ brain	70%
	*Firre*^+/+^ liver	67%
	*Firre*^−/−^ liver	69%
	*Firre*^+/+^ kidney	56%
	*Firre*^−/−^ kidney	58%

The table lists each cell line, including its origin, its genotype, and the percentage of nuclei with a H3K27me3 cluster on the Xi.

Next, allele-specific profiles of H3K27me3 were generated by ChIP-seq, which demonstrated a chromosome-wide decrease on the Xi in Δ*Firre*^Xa^ (Fig. 2d). The loss of H3K27me3 in mutant cells was quantified by counting unique reads mapped to the Xa, Xi, and autosomes. In WT the Xi/Xa read ratio was 2.45, reflecting the characteristic H3K27me3 enrichment on the Xi. In contrast, this ratio was only 1.60 in Δ*Firre*^Xa^, representing a significant decrease on the Xi (Fig. 2d). The BL6/*spretus* read ratios for autosomes in WT and Δ*Firre*^Xa^ remained similar, 1.04 and 1.14, respectively. The 34% percent decrease in the level of H3K27me3 on the Xi as measured by ChIP-seq is lower than that measured by immunostaining (89%), which is expected based on known differences between methodologies. While immunostaining captures condensation of the Xi and thus results in many nuclei with a visible H3K27me3 cluster, ChIP-seq reveals uneven distribution of H3K27me3 along the Xi and thus an apparently lesser enrichment^37^. In fact, the 34% change in H3K27me3 we measured by ChIP-seq in Δ*Firre*^Xa^ versus WT is similar to that reported between undifferentiated and differentiated mouse ES cells, in which the onset of XCI causes only a 40% increase in H3K27me3 level as measured by ChIP-seq, while over 90% of nuclei acquire a visible H3K27me3 cluster, as determined by immunostaining^37^.

Next, we calculated allelic proportions of SNP read coverage for each H3K27me3 peak covered by at least five SNP reads. In both WT and Δ*Firre*^Xa^ the distribution of allelic proportions (*spretus*/(*spretus* + BL6)) for the autosomes centered close to the anticipated 0.5, reflecting a similar enrichment between alleles (Fig. 2e). In contrast, the distribution of allelic proportions for the X chromosomes (Xa/(Xa + Xi)) centered at ~0.35 in WT, consistent with H3K27me3 enrichment on the Xi, but was markedly shifted to higher values (~0.5) in Δ*Firre*^Xa^, supporting the 34% decrease in H3K27me3 on the Xi (Fig. 2e). Heatmaps and metagene plots of allelic ChIP-seq data further demonstrate a dramatic loss of H3K27me3 around the transcription start site and throughout the body of X-linked genes in mutant cells (Fig. 2f, g). LINE and SINE repeats also show lower H3K27me3 on the Xi in Δ*Firre*^Xa^ (Supplementary Fig. 2c). Finally, we confirmed a decrease in H3K27me3 in Δ*Firre*^Xa^ using CUT&RUN in a separate study^38^.

Next, we investigated SUZ12, a subunit of the PRC2 complex, using CUT&RUN, which showed a decrease on the Xi in Δ*Firre*^Xa^. Again, the distribution of allelic proportions (Xa/(Xa+Xi)) for each SUZ12 peak showed a pronounced shift toward higher values (~0.75) for the X chromosomes in Δ*Firre*^Xa^ versus WT, while allelic proportions for the autosomes (0.5) did not change (Fig. 2h). Reanalysis of published datasets of RNA/protein interactions in mouse cells confirmed *Firre* RNA interactions with PRC components. Analysis of RIP-seq data showed *Firre* RNA interactions with two components of PRC2, EZH2 and SUZ12, and with hnRNPK, a protein that recruits the noncanonical PCGF3/5-PRC1 (Fig. 2i; Supplementary Data 5)^39,40^. Analysis of data obtained by CLIP-seq and PAR-CLIP, two UV-crosslinking-based methods for highly accurate mapping of RNA-protein interactions, confirmed *Firre* RNA interactions with EZH2 and detected interactions with JARID2 and RBFOX2, two proteins known to help recruit PRC2 to chromatin, and with CBX7, a component of the canonical PRC1 complex implicated in H3K27me3 deposition^41–45^ (Supplementary Data 5).

We conclude that *Firre* RNA transcribed from the Xa specifically helps target PRC1 and PRC2 complexes to the Xi for maintenance of H3K27me3, consistent with a trans-acting effect in Patski cells. In support we find that several proteins implicated in the PRC complexes are *Firre* RNA interactors.

### Dose-dependent and cell-type-specific effects of *Firre*

**Fig. 3:**
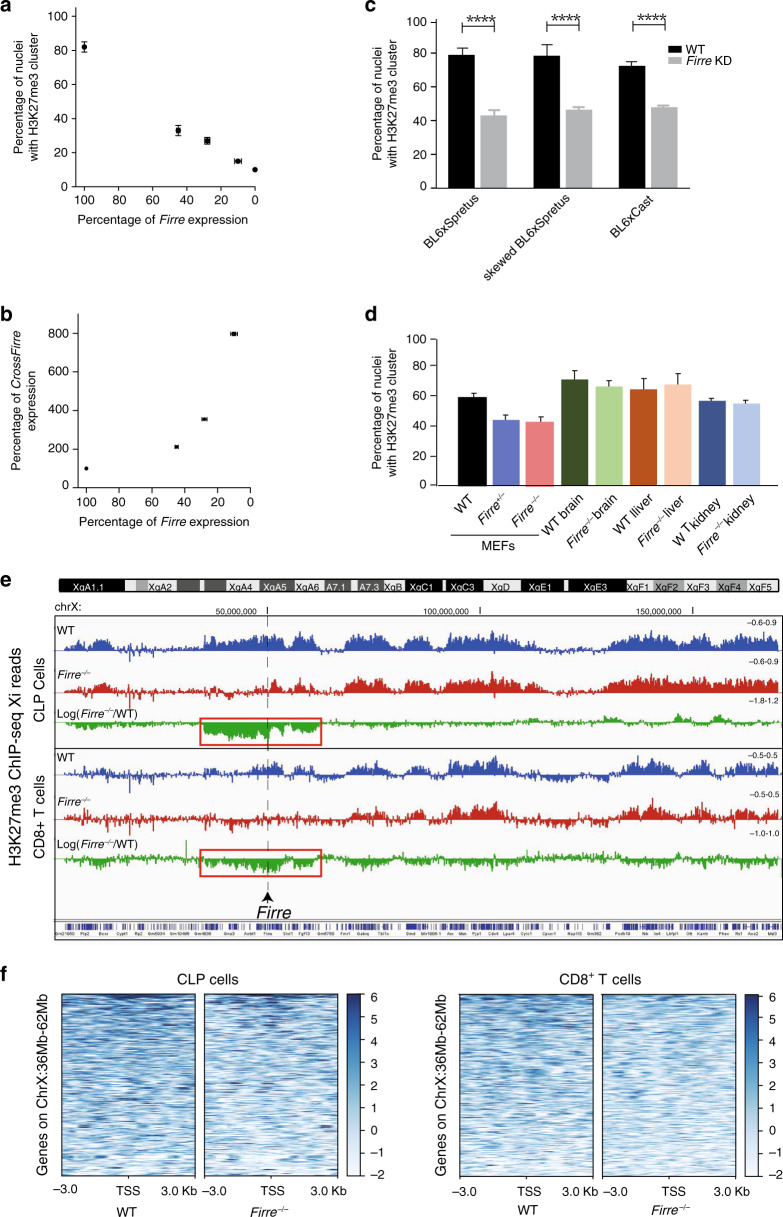
Dose-dependent effects of *Firre* RNA on H3K27me3 on the Xi in cell lines and in vivo. **a** Percentage of nuclei with a H3K27me3 cluster after KD in Patski cells relative to *Firre* expression level measured by qRT-PCR in *n* = 3 biologically independent samples. Data presented as mean values ± SEM. **b** The level of *CrossFirre* expression is inversely related to that of *Firre* after *Firre* KD. Expression measured by qRT-PCR in *n* = 3 biologically independent samples. Data presented as mean values ± SEM. **c, d** A total of >300 Patski nuclei were scored per cell type over three independent experiments; significance was determined by one-sided Fisher exact test; bar plots are presented as mean values ± SEM; scale bars represent 10 µm. **c** Bar plots show significantly fewer H3K27me3 clusters after *Firre* KD in nuclei from three primary MEFs compared to mock treatment (*p* value = 2.89075e-12, 1.41519e-32, and 8.95838e-10, respectively). Primary MEFs were derived from a (BL6 × *spretus*) embryo with a *spretus* Xi, and from embryos either (BL6 × *spretus*) or (BL6 × *castaneus*) with random XCI (Table1). **d** Bar plots show a lower (but not significantly) percentage of H3K27me3 cluster in nuclei from MEFs derived from *Firre*^+/−^ and *Firre*^−/−^ KO embryos than in controls (*p* value = 0.0774 and 0.0567, respectively). No significant differences were found in brain, liver, and kidney from *Firre* KO mice compared to WT (*p* value = 0.9127, 0.8796, and 0.762, respectively). **e** Profiles of H3K27me3 ChIP-seq reads along the X chromosomes from CLPs and CD8 + T cells from WT (blue), *Firre*^−/−^ (red), and log_2_ ratio *Firre*^−/−^/WT (green) show a significant decrease covering ~26 Mb around the *Firre* locus in mutant CLPs, and to a lesser extent CD8 + T cells (Supplementary Data 6). **f** Heatmaps of H3K27me3 ChIP-seq reads located 3 kb around the transcription start sites (TSS) of X-linked genes that map within the 26 Mb region around *Firre* in WT and *Firre*^−/−^ CLPs and CD8 + T cells.

To quantify the effects of *Firre*, we knocked it down in a dose-dependent manner using shRNA and siRNA treatments of an independent Patski isolate to achieve KD levels ranging from 43, 28, and 10% of WT. This caused a parallel reduction in percentages of nuclei with a H3K27me3 cluster to 33%, 27%, and 15%, respectively, versus 83% in WT (*p* < 0.0001) (Fig. 3a; Table 1). Importantly, this reveals a dose-dependent effect of *Firre* RNA on H3K27me3 enrichment on the Xi (Fig. 3a). Concomitantly, a dose-dependent increase in *CrossFirre* RNA inversely correlates to the amount of *Firre* RNA, consistent with *Firre* repressing the antisense transcript (Fig. 3b).

A separate KD to deplete *Firre* RNA to 33% of WT in primary mouse embryonic fibroblasts (MEFs) independently derived from a BL6 × *spretus* F1 embryo with random XCI significantly decreased the percentage of nuclei with a H3K27me3 cluster to 43%, versus 79% in controls (*p* < 10^−4^) (Fig. 3c; Table 1). To exclude species-specific effects, we tested MEFs from a BL6 × *spretus* F1 embryo with an XCI pattern opposite to that in Patski cells (i.e., the Xi is from *spretus*), and MEFs from a BL6 × *castaneus* F1 embryo. After KD in these lines *Firre* RNA level was lowered to 21 and 15% of WT, causing a significant reduction to 45 and 47% of nuclei with a H3K27me3 cluster, compared to 80 and 78% in controls, respectively (*p* < 10^−4^) (Fig. 3c; Supplementary Fig. 2d; Table 1). No significant change in *CrossFirre* was found in those lines (Supplementary Fig. 2e). We then examined cells and tissues from a *Firre* KO mouse model^32,33^. MEFs from heterozygous (*Firre*^+/−^) and homozygous (*Firre*^−/−^) KO female embryos showed a modest reduction to 43 and 41% of nuclei with a H3K27me3 cluster, respectively, compared to 58% in a control littermate (*Firre*^+/+^) (Fig. 3d; Table 1). H3K27me3 immunostaining in liver, kidney, and brain sections derived from *Firre*^−/−^ female mice showed no significant decrease in nuclei with a cluster (Fig. 3d; Table 1).

Next, we performed H3K27me3 ChIP-seq analysis of sorted CLPs and CD8 + T cells derived from *Firre*^−/−^ KO female mice. Surprisingly, we found a striking loss of H3K27me3 in a ~26 Mb (ChrX: 36–62 Mb) region centered around the *Firre* locus, together with a slight decrease across the entire X chromosome, but not the autosomes (Fig. 3e, Supplementary Fig. 3a, b). Heatmaps of control and *Firre*^−/−^ KO CLPs and CD8 + T cells showed a loss of H3K27me3 around the transcription start site of genes located within this 26 Mb region (Fig. 3f, Supplementary Data 6). Although we assume that this local loss of H3K27me3 affects the Xi and not the Xa, allelic analysis was not possible since KO mice are homozygous BL6. The 26 Mb region around *Firre* contains 266 curated genes, 15 involved in immunity (e.g., *Nkap, Sash3, Atp11c*, *Sh2d1a*, *Elf4*^46–48^), and 15 potentially exhibiting haploinsufficiency effects in human (e.g., *GPC3*^49^) (Supplementary Data 6). However, no specific expression changes have been reported for any of these genes in CLPs from KO mice^33^.

In summary, we found a dose-dependent effect of *Firre* RNA in maintenance of H3K27me3 on the Xi in several types of fibroblasts. Lack of detectable changes in three tissues from *Firre* KO mice suggests that the role of *Firre* may be cell-type- and tissue-specific, as proposed for other lncRNAs^50^. Consistent with the role of *Firre* in hematopoiesis, local changes were observed in CLPs and CD8 + T cells around the *Firre* locus, revealing a cell-type-specific role in cis-maintenance of H3K27me3^33^.

### Rescue of H3K27me3 on the Xi by *Firre/FIRRE* cDNA transgenes

**Fig. 4:**
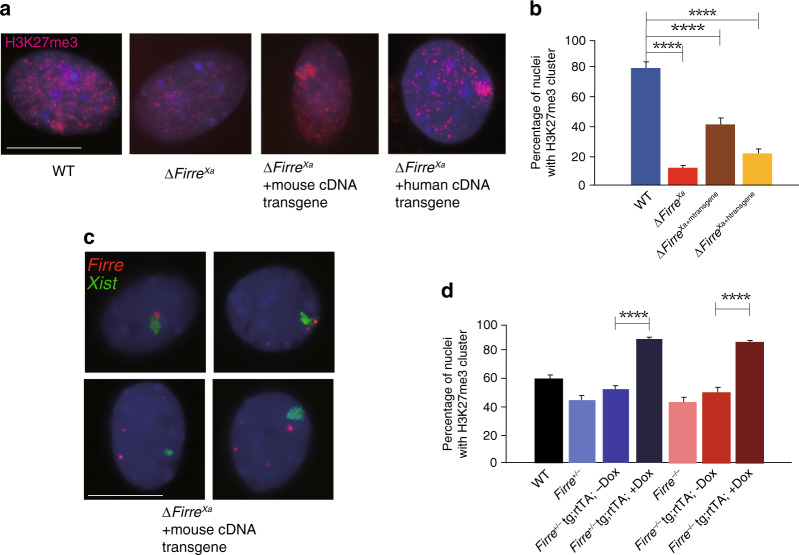
Ectopic expression of *Firre/FIRRE* RNA partially restore H3K27me3 on the Xi. **a** Examples of nuclei after H3K27me3 immunostaining (red) and Hoechst 33342 staining (blue) in WT, Δ*Firre*^Xa^, and Δ*Firre*^Xa^ transfected with a mouse *Firre* transgene (Δ*Firre*^Xa+mtransgene^) or a human *FIRRE* transgene (Δ*Firre*^Xa+htransgene^). **b** The bar plot shows a significantly higher percentage of nuclei with a H3K27me3 cluster in cells with a mouse or human *Firre/FIRRE* transgene, compared to Δ*Firre*^Xa^ (*p* value = 2.8075e-16 and 0.0000134599, respectively). **c** Examples of nuclei after RNA-FISH for *Xist* (green) and *Firre* (red) in a Δ*Firre*^Xa+mtransgene^ cell clone with high expression of the mouse transgene. The upper two nuclei show association between *Firre* and *Xist* signals (seen in 15% of nuclei), and the lower nuclei, lack of association. **d** The bar plot shows the percentage of nuclei with a H3K27me3 cluster in MEFs derived from *Firre*^+/−^ and *Firre*^−/−^ females that harbor a doxycycline (Dox) inducible transgene (*Firre*^+/−^ tg;rtTA; -Dox; *Firre*^+/−^ tg;rtTA; +Dox; *Firre*^−/−^ tg;rtTA; -Dox; *Firre*^−/−^ tg;rtTA; +Dox), compared to WT and mutants. The percentage of nuclei with a H3K27me3 cluster increases significantly in *Firre*^+/−^ tg;rtTA and *Firre*^−/−^ tg;rtTA MEFs after addition of doxycycline (Dox+) (p value=4.31567e-30 and 3.51193e-20, respectively). A total of >300 nuclei scored for the presence of a H3K27me3 cluster (**a**, **b, d**), or for *Xist* and *Firre* RNA-FISH signals (**c**) per cell type over three independent experiments; significance was determined by one-sided Fisher exact test; bar plots are presented as mean values ± SEM; scale bars represent 10 µm.

To confirm a causative role of *Firre* RNA on H3K27me3 enrichment on the Xi, Δ*Firre*^Xa^ cells were transfected with a mouse *Firre* cDNA transgene that lacks the first five 5’ exons of *Firre* and is expressed from a CMV promoter (Supplementary Fig. 1b). By RNA-seq *Firre* expression was restored to a near-normal level after transfection. Ectopic expression of the mouse cDNA in Δ*Firre*^Xa+mtransgene^ cells rescued the presence of a H3K27me3 cluster from 9 to 38% of nuclei, supporting a trans-acting role (Fig. 4a, b, Table 1). This partial rescue is not readily explained by heterogeneous transgene levels among cells, since a cloned transgenic line with stable high *Firre* expression rescued to a similar level (34%). *Firre* RNA-FISH in this clone showed association of the lncRNA to the *Xist* cloud in 15% of cells, consistent with partial rescue (Fig. 4c). Incomplete rescue is likely due to the partial cDNA transgene that may lack functional RNA motifs, isoforms, and regulatory elements (Supplementary Fig. 1b). Interestingly, we also observed partial rescue after ectopic expression of a human *FIRRE* cDNA in Δ*Firre*^Xa+htransgene^ cells (Fig. 4a, b; Table 1). Next, we examined *Firre*^+/−^ and *Firre*^−/−^ MEFs from KO mice with an ectopic doxycycline (DOX)-inducible plasmid copy of a complete *Firre* cDNA integrated in the genome. After *Firre* expression induction (10–20 fold) a strong increase to 91 and 83% of nuclei with a H3K27me3 cluster was found compared to 43 and 41% in noninduced KO cells, respectively (Fig. 4d; Table 1).

We conclude that loss of H3K27me3 on the Xi in mutant cells is rescuable by a cDNA transgene, supporting *Firre* trans-acting role. Surprisingly, a human *FIRRE* transgene can also rescue to some extent, suggesting functional compatibility between species despite sequence divergence. Results of induction of a transgene in the mouse KO model further support a dose-dependent trans-effect of *Firre* RNA on H3K27me3 enrichment on the Xi.

### *Firre* acts in trans to maintain nuclear location of the Xi

**Fig. 5:**
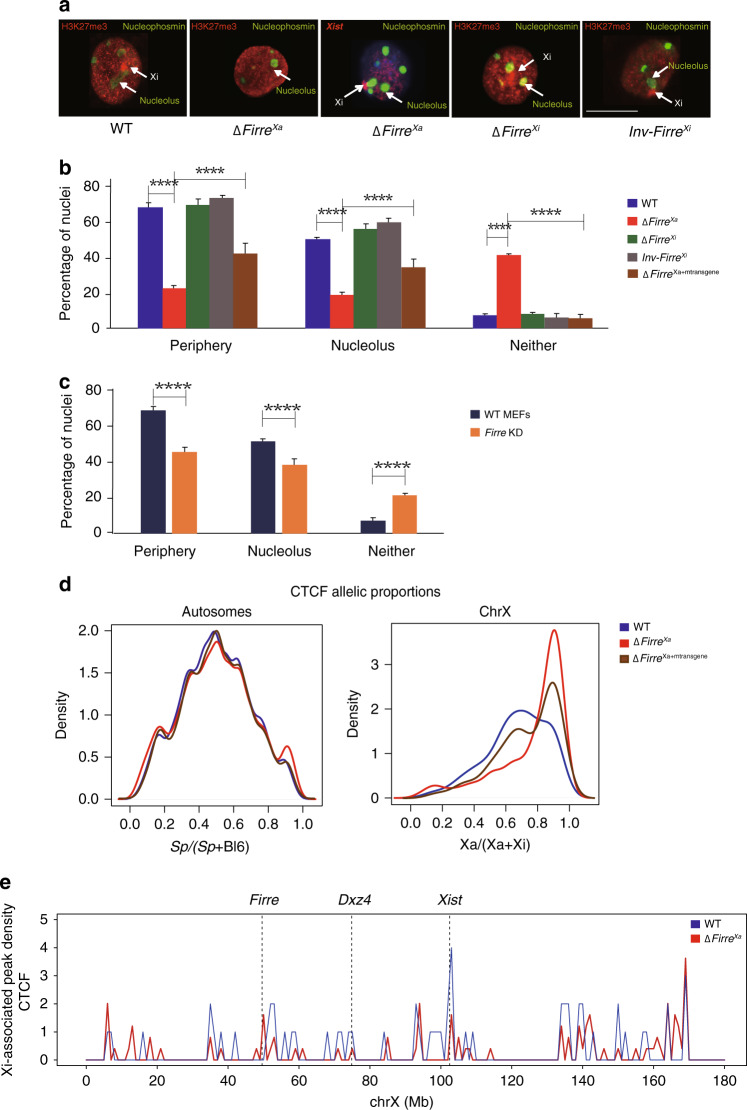
*Firre* RNA acts in trans to maintain Xi location. **a**–**c** A total of >300 nuclei per cell type over 3 independent experiments were scored for the location of the Xi marked by a H3K27me3 cluster or an *Xist* cloud relative to the nuclear periphery or the nucleolus; significance was determined by one-sided Fisher exact test; bar plots are presented as mean values ± SEM; scale bars represent 10 µm. **a** Examples of nuclei after H3K27me3 immunostaining (red) to locate the Xi in WT, Δ*Firre*^Xi^, and Inv*Firre*^Xi^, or *Xist* RNA-FISH (red) to locate the Xi in Δ*Firre*^Xa^ since there is no H3K27me3 cluster in these nuclei. Nuclei were also immunostained for NPM1 (green) to locate the nucleolus. **b** Bar plots show a significant decrease in periphery- and nucleolus association of the Xi in Δ*Firre*^Xa^ compared to WT (*p* value = 3.95632e-40 and 3.75497e-14, respectively), but no significant changes in Δ*Firre*^Xi^ and Inv*Firre*^Xi^ (*p* value = 0.6968). Ectopic expression of a mouse transgene in Δ*Firre*^Xa+mtransgene^ partly rescues the Xi location to 41% at the periphery and 34% at the nucleolus (*p* value = 6.62701e-10 and 0.000265481, respectively). **c** Bar plots show a significant decrease in periphery- and nucleolus association of the Xi after *Firre* KD in primary MEFs derived from an F1 embryo (BL6 × *spretus*) (*p* value = 1.13757e-09 and 0.0000634923, respectively). **d** Density histograms of the distribution of allelic proportions (Xa/(Xa + Xi)) of CTCF peaks show a shift in the distribution for the X chromosomes due to a decrease in CTCF on the Xi in Δ*Firre*^Xa^ (red) compared to WT (blue). In Δ*Firre*^Xa+mtransgene^ (brown) this distribution becomes binomial due to partial restoration of CTCF on the Xi. **e** Plots of Xi-associated (common +Xi-specific) CTCF peak density (counts binned within 100 kb windows) along the Xi for WT (blue) and Δ*Firre*^Xa^ (red). To account for differences in the number of SNP-covered peaks due to sequencing depth, the binned counts are scaled by a factor obtained from the between-sample ratios of autosomal diploid SNP-covered peaks.

We next examined the effects of allelic *Firre* mutations on the Xi location relative to the nucleolus and lamina. In WT, Δ*Firre*^Xi^, and Inv*Firre*^Xi^ H3K27me3 and nucleophosmin (NPM1) immunostaining was used to locate the Xi and the nucleoli, respectively (Fig. 5a). Since the H3K27me3 cluster is compromised in Δ*Firre*^Xa^, *Xist* RNA-FISH was applied in combination with NPM1 immunostaining. The Xi location was scored in WT nuclei as adjacent to the nuclear periphery (70%), the nucleolus surface (50%), or neither (8%) (Fig. 5b). Note that in 28% of nuclei the Xi was close to both the periphery and the nucleolus. Loss of *Firre* RNA in Δ*Firre*^Xa^ caused significant reductions in Xi-nuclear periphery and Xi-nucleolus associations to 20 and 22% of nuclei, respectively (*p* < 0.0001) (Fig. 5b). Importantly, ectopic expression of a cDNA transgene in Δ*Firre*^Xa+mtransgene^ significantly (but not completely) rescued these associations (Fig. 5b). Deletion or inversion of *Firre* on the Xi did not alter its location (Fig. 5a, b). In primary MEFs independently derived from a BL6 × *spretus* F1 mouse with random XCI *Firre* KD also caused a decrease in Xi-nuclear periphery and Xi-nucleolus associations (Fig. 5c).

CTCF has been implicated in nucleolus association of genomic regions, and we have previously shown that *Ctcf* KD reduces Xi-nucleolus associations in Patski cells^20,51^. Here, we profiled allelic CTCF binding by CUT&RUN, which demonstrated a loss of CTCF binding on the Xi in Δ*Firre*^Xa^ (Fig. 5d). The distribution of allelic proportions (Xa/(Xa + Xi)) for CTCF peaks on the X chromosomes showed a pronounced shift toward higher values (~0.85) in Δ*Firre*^Xa^ compared to WT (~0.65), while allelic proportions (*spretus*/(*spretus* + BL6)) for the autosomes were close to the anticipated 0.5 (Fig. 5d). Thus, while there is less CTCF binding on the Xi versus Xa in WT as expected, CTCF binding is even lower in Δ*Firre*^Xa^ (Fig. 5e). In Δ*Firre*^Xa+mtransgene^ the distribution of allelic proportions for the X chromosomes becomes binomial, indicating partial restoration of CTCF on the Xi (Fig. 5d).

Taken together, our results show that *Firre* RNA transcribed from the Xa or from an ectopic cDNA transgene can influence in trans the Xi location within the nucleus. Our results further suggest a potential cooperation between *Firre* RNA and CTCF in maintenance of Xi location.

### Loss of *Firre* affects gene expression in Patski cells

**Fig. 6:**
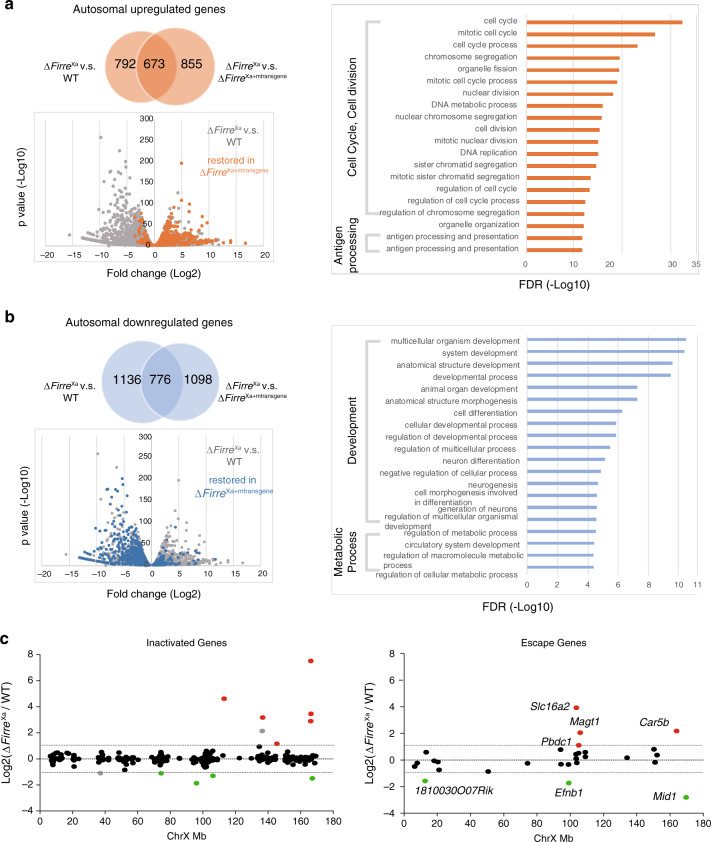
Loss of *Firre* RNA affects gene expression. **a** Upregulated genes in Δ*Firre*^Xa^ and Gene Ontology (GO) term enrichment. The Venn diagram shows the number of upregulated genes in Δ*Firre*^Xa^ versus WT, and in Δ*Firre*^Xa^ versus Δ*Firre*^Xa+mtransgene^, with the overlapping gene set representing upregulated genes in Δ*Firre*^Xa^ that are rescued by transgene expression. The scatter plot shows dysregulated genes in Δ*Firre*^Xa^ versus WT (grey), with genes rescued by reduced expression in Δ*Firre*^Xa+mtransgene^ versus Δ*Firre*^Xa^ (more than 2-fold; *p* value < 0.05 by the Wald test) highlighted in orange. The top 20 GO terms of overlapping upregulated genes in Δ*Firre*^Xa^ versus WT, which are rescued in Δ*Firre*^Xa+mtransgene^ are listed. The *X*-axis indicates the FDR (−log10). **b** Downregulated genes in Δ*Firre*^Xa^ and Gene Ontology (GO) term enrichment. The Venn diagram shows the number of downregulated genes in Δ*Firre*^Xa^ versus WT, and in Δ*Firre*^Xa^ versus Δ*Firre*^Xa+mtransgene^, the overlapping gene set representing downregulated genes in Δ*Firre*^Xa^ that are rescued by transgene expression. The scatter plot shows dysregulated genes in Δ*Firre*^Xa^ versus WT (gray), with genes rescued by increased expression in Δ*Firre*^Xa+mtransgene^ versus Δ*Firre*^Xa^ (more than 2-fold; *p* value <0.05 by the Wald test) highlighted in blue. The top 20 GO terms of overlapping downregulated genes in Δ*Firre*^Xa^ versus WT, which are rescued in Δ*Firre*^Xa+mtransgene^ are listed. The *X*-axis indicates the FDR (−log10). **c** Xi-expression fold changes for genes that are subject to or escape XCI between Δ*Firre*^Xa^ and WT. Upregulated genes are in red and downregulated genes in green. To note, one upregulated gene and one gene subject to XCI are in gray, as they showed more than 2-fold change in expression but with a *p* value >0.05 by the Wald test in Δ*Firre*^Xa^ versus WT. Genes are ordered from centromere to telomere along the Xi.

Next, we examined changes in total gene expression (autosomal and X-linked genes without allele discrimination) in Δ*Firre*^Xa^. About 11 and 14% of genes with expression ≥1TPM in at least one condition were upregulated and downregulated in Δ*Firre*^Xa^ versus WT, respectively, (Supplementary Data 7 and 8). A large proportion (46 and 40%, respectively) of dysregulated genes were rescued in Δ*Firre*^Xa+mtransgene^ (Fig. 6a, b, Supplementary Fig. 4a, Supplementary Data 7). To rule out effects of aneuploidy between cell lines results were confirmed for genes located on diploid chromosomes in WT and Δ*Firre*^Xa^ (Supplementary Fig. 4b, Supplementary Data 9). GO analysis show that the top 20 GO terms for genes upregulated in Δ*Firre*^Xa^ and rescued in Δ*Firre*^Xa+mtransgene^ are related to cell cycle, DNA replication, chromosome segregation, and immune cell function, while downregulated genes are implicated in development, differentiation, and metabolism (Fig. 6a, b; Supplementary Data 10). In human HEK293 cells *FIRRE* RNA KD resulted in dose-dependent cell death, consistent with a role in cell growth and survival (Supplementary Fig. 4c).

To determine whether gene expression was disrupted on the Xi upon loss of *Firre* RNA we evaluated allelic gene expression. Only 6/352 genes known to be subject to XCI showed >2-fold upregulation in Δ*Firre*^Xa^, suggesting minor Xi reactivation (Fig. 6c, Supplementary Data 3 and 7). Four of the reactivated genes were located at the telomeric end of the Xi where higher accessibility and decreased contact density were observed by ATAC-seq and Hi-C (see below). Although their number is small and we cannot rule out a chance occurrence, more genes known to escape XCI were dysregulated (10–14%) from the Xi than genes subject to XCI (2–3%) (Fig. 6c, Supplementary Fig. 4d, Supplementary Data 7). A majority of dysregulated X-linked genes (67%) were rescued in Δ*Firre*^Xa+mtransgene^ (Supplementary Fig. 4e, Supplementary Data 3 and 7).

Metagene plots of H3K27me3 along autosomal and X-linked genes grouped based on their expression changes in Δ*Firre*^Xa^, together with profiles of individual genes showed a small increase in H3K27me3 for downregulated genes, and very little or no change for upregulated or unchanged genes (Supplementary Fig. 5a, b). Note that 90% of dysregulated genes have no H3K27me3 enrichment in either WT or Δ*Firre*^Xa^. Additional CUT&RUN analyses of two active epigenetic marks, H3K36me3 and H3K4me3, showed no significant changes on the Xi in Δ*Firre*^Xa^, consistent with little Xi reactivation (Supplementary Fig. 5c, d)

Thus, the loss of *Firre* RNA causes widespread changes in gene expression in large part rescued by a cDNA transgene. Very little reactivation and few changes in escape genes occur on the Xi despite the observed loss of H3K27me3, consistent with this epigenetic mark representing only one layer of XCI control^20^.

### Allelic alterations of the *Firre* locus change Xi structure

**Fig. 7:**
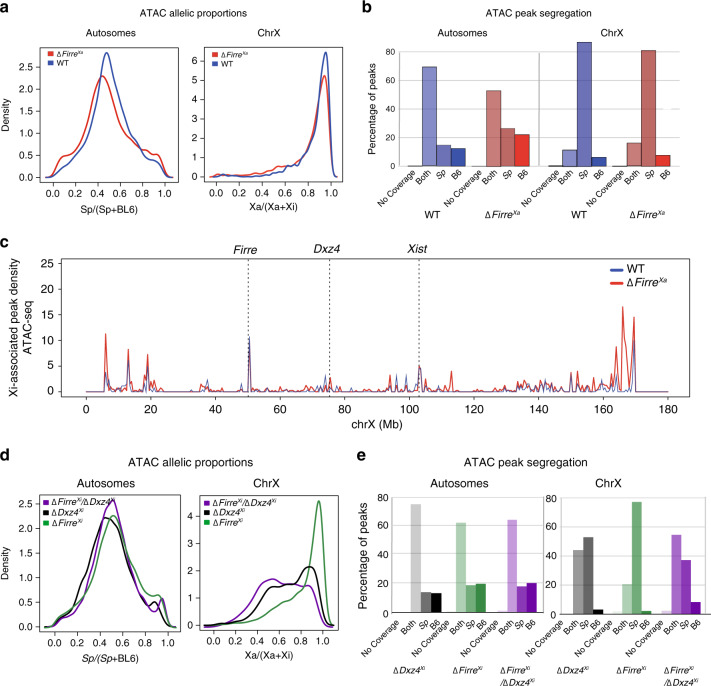
Chromatin accessibility after allelic *Firre* deletions and a *Firre/Dxz4* deletion. **a** Density histograms of the distribution of allelic proportions (*spretus*/(*spretus* + BL6)) of ATAC peaks along the autosomes and the X chromosomes for WT (blue) and Δ*Firre*^Xa^ (red). No shift is observed (Wilcoxon test: −log10P = 32). **b**. Percentages of ATAC peaks along the autosomes and the X chromosomes classified as *spretus*-specific, BL6-specific, or both show no differences between WT (blue) and Δ*Firre*^Xa^ (red). **c**. Plots of Xi-associated (common +Xi-specific) ATAC peak density (counts binned within 500 kb windows) along the Xi show increased accessibility at the telomeric end of the Xi in Δ*Firre*^Xa^ (red) versus WT (blue). To account for differences in the number of SNP-covered peaks between samples due to sequencing depth, the binned counts are scaled by a factor obtained from the between-sample ratios of autosomal diploid SNP-covered peaks. **d** Density histograms of the distribution of allelic proportions (*spretus*/(*spretus* + BL6)) of ATAC peaks show a shift to a lower Xa/(Xa + Xi) ratio in the double-mutant Δ*Firre*^Xi^/Δ*Dxz4*^Xi^ (purple), compared to Δ*Dxz4*^Xi^ (black) and Δ*Firre*^Xi^ (green), consistent with increased accessibility on the Xi (Wilcoxon test: −log10P = 35). **e** Percentages of ATAC peaks in Δ*Dxz4*^Xi^ (black), Δ*Firre*^Xi^ (green), and Δ*Firre*^Xi^/Δ*Dxz4*^Xi^ (purple) along the autosomes and the X chromosomes classified as *spretus*-specific, BL6-specific, or both show an increase on the BL6 Xi in the double mutant.

We evaluated chromatin accessibility in *Firre* mutants by ATAC-seq. As expected, in WT the distribution of allelic proportions centered at 0.5 for autosomes, but was skewed towards the Xa (0.95) for the X chromosomes, indicative of lower chromatin accessibility on the Xi. This pattern remained similar in Δ*Firre*^Xa^, consistent with near absence of gene reactivation. Plots of ATAC peak density further captured the low accessibility profiles of the Xi in both WT and Δ*Firre*^Xa^ (Fig. 7a, b). However, a higher peak density was observed in the telomeric region of the Xi in Δ*Firre*^Xa^ where reactivated genes are located (Fig. 6c; 7c). ATAC-seq patterns were unchanged in Δ*Firre*^Xi^ and Inv*Firre*^Xi^ (Supplementary Fig. 6a, b). To determine whether *Dxz4* and *Firre* may have synergistic cis-effects on chromatin accessibility on the Xi, ATAC-seq was done on a double-mutant line Δ*Firre*^Xi^/Δ*Dxz4*^Xi^. Interestingly, a pronounced shift to lower values in the distribution of allelic proportions for the X chromosomes (peak at ~0.55) was observed in this double-mutant compared to either Δ*Dxz4*^Xi^ (peak at ~0.85) or Δ*Firre*^Xi^ (peak at ~1), indicating increased chromatin accessibility on the Xi (Fig. 7d, e).

**Fig. 8:**
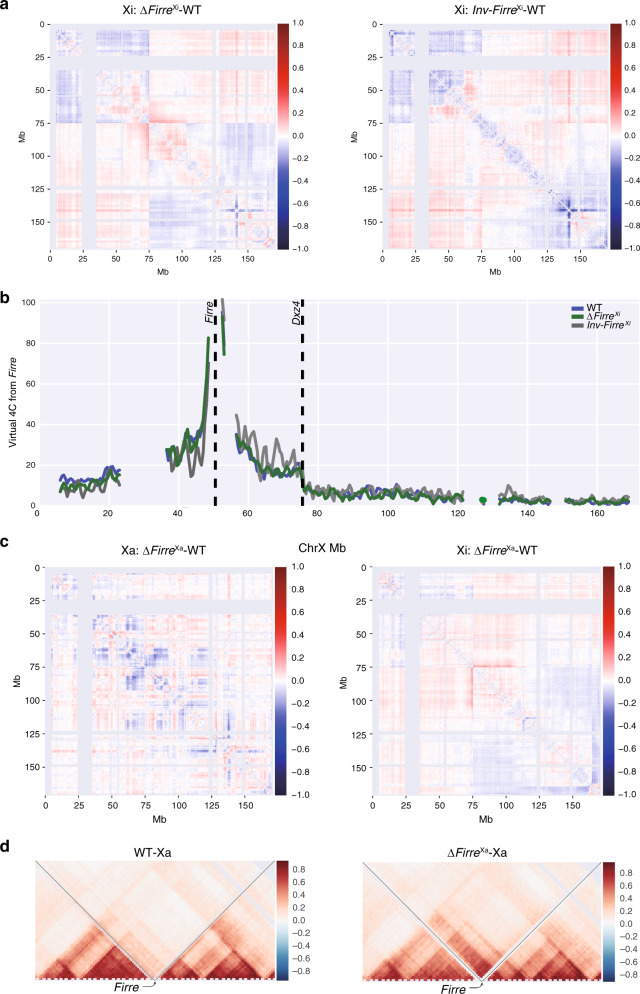
Changes in the X 3D structure after *Firre* deletion or inversion. **a** Pearson correlated-transformed differential contact maps of the Xi at 500 kb resolution highlight differences between Δ*Firre*^Xi^ and WT, and between Inv*Firre*^Xi^ and WT. The color scale shows differential Pearson correlation values, with loss and gain of contacts in the mutants versus WT appearing blue and red, respectively. See text for description of changes. **b** Virtual 4 C plots derived from Hi-C data at 500 kb resolution using *Firre* as the viewpoint on the Xi in WT (blue), Δ*Firre*^Xi^ (green) and Inv*Firre*^Xi^ (gray) show an increase in contacts between *Firre* and *Dxz4* in Inv*Firre*^Xi^. **c** Pearson correlated-transformed differential contact maps of the Xa and Xi at 500 kb resolution to highlight differences between Δ*Firre*^Xa^ and WT. The color scale shows differential Pearson correlation values, with loss and gain of contacts in Δ*Firre*^Xa^ versus WT appearing blue and red, respectively. See text for description of changes. **d** Pearson correlated-transformed contact maps (40 kb resolution) of the Xa for 4 Mb around the *Firre* locus highlight the loss of the strong boundary between TADs on the Xa in Δ*Firre*^Xa^ versus WT (see Supplementary Fig. 7c for corresponding maps of the Xi).

Based on high-resolution allele-specific contact maps generated by DNase Hi-C the Xi bipartite structure was retained in Δ*Firre*^Xi^, but contacts increased between the superdomains and decreased within each superdomain, suggesting that the *Firre* locus acts in cis to help shape the 3D structure (Fig. 8a). Contacts within regions flanked by *Dxz4* and *Xist* (ChrX:75–100 Mb) and to a lesser extent, flanked by *Firre* and *Dxz4* (ChrX:50–75 Mb) were increased, suggesting disruption of contacts emanating from *Dxz4* (Fig. 8a, Supplementary Fig. 7a). Similarly, Inv*Firre*^Xi^ nuclei showed persistence of the Xi bipartite structure, but also a redistribution of contacts around *Firre*, including loss of proximal contacts (ChrX:5–50 Mb) and gain of distal contacts (ChrX:50–75 Mb), which was confirmed by virtual 4 C (Fig. 8a, b; Supplementary Fig. 7a). The boundary at or near the *Firre* locus on the WT Xi was maintained upon deletion or inversion of the locus (Supplementary Fig. 7b). While the loss of *Firre* RNA in Δ*Firre*^Xa^ did not perturb the bipartite structure of the Xi, there were some changes in contact distribution, suggesting trans-effects (Fig. 8c, Supplementary Fig. 7c). Specifically, contacts increased in the region flanked by *Dxz4* and *Xist* (ChrX:75–100 Mb), and diminished in the very distal telomeric region (ChrX:165–170 Mb), consistent with an increase in chromatin accessibility and gene expression (Figs. 6c, 7c, 8c). Deletion of *Firre* on the Xa also resulted in contact changes on the Xa, including loss of the boundary at or close to the locus, as confirmed by insulation score analysis (Fig. 8d; Supplementary Fig. 7d, e).

Taken together, our results indicate cooperation between *Firre* and *Dxz4* in repression of chromatin accessibility on the Xi, with each locus contributing to the two superdomains separation. *Firre* contacts with other regions on the Xi appear orientation-dependent, reminiscent to the orientation-dependent contacts made by *Dxz4*^29^. *Firre* RNA exerts trans-effects on the Xi 3D structure, potentially secondary to losses of H3K27me3 and CTCF binding.

## Discussion

Studies of lncRNAs support the notion that these molecules can either spread in cis from their genomic locus or localize to cellular compartments away from their own locus of transcription to perform essential functions in regulating gene expression^52–55^. Here, we report that the lncRNA *Firre* transcribed from the Xa acts in trans and in cis on the Xi to maintain its epigenetic features, nuclear location, and 3D structure. LncRNAs have important roles in the structure of nuclei where they fold into higher-order structures and act in cooperation with proteins including chromatin-modifying complexes^56^. *Xist* represents the quintessential example of a lncRNA that spreads along the Xi in cis to recruit a series of proteins including components of the PRC complexes that implement chromatin modifications such as H3K27me3^2,3,6,19,56^. We find that maintenance of H3K27me3 on the Xi mediated by *Firre* RNA involve the PRC complexes, which is supported by our reanalyses of RNA/protein interaction datasets^39–45^. Among *Firre* interactors EZH2 and SUZ12 represent core subunits of PRC2, while JARID2 and RBFOX2 are cofactors that directly interact with RNA^57–59^. PRC2 recruitment of the noncanonical PCGF3/5-PRC1 complex is facilitated by the *Firre* interactor hnRNPK^60^, while CBX7 is a subunit of the canonical PRC1 complex^61^. A new method to detect protein-RNA interactions (incPRINT) also identifies JARID2, EPC1, CTCF, and hnRNPU as *Firre* interactors^62^. Most *Firre* interactors have previously been implicated in XCI, and more specifically in H3K27me3 enrichment on the Xi (Supplementary Data 5)^2,6,63–66^. Colocalization of *Firre* RNA to the Xi was seen in only 15% of nuclei, suggesting dynamic binding of the lncRNA. Interestingly, single particle tracking of endogenous EZH2 and SUZ12 in human cells reveals rapid diffusion of PRC2 through the nucleus, with only ~20% chromatin-bound^67^.

Trans-effects have been reported for several lncRNAs. For example, *Fendrr* and *Pint* recruit PRC2 for H3K27 tri-methylation of loci located on other chromosomes^68,69^. *Meg3* also recruits PRC2 components, JARID2 and EZH2, to facilitate H3K27me3 deposition and repression of genes in trans^42,70^. *Meg3* has an additional cis-acting role by sequestration of PRC2 to prevent DNA methylation-induced repression of genes within the *Meg3-Mirg* imprinting cluster^71,72^. Interestingly, we observe a local loss of H3K27me3 centering around the *Firre* locus and extending to 26 Mb in CLPs and CD8 + T cells from *Firre* KO female mice, suggesting that *Firre* may also act in cis to maintain H3K27me3 in certain cell types. Importantly, defects in hematopoiesis that impact CLPs have been observed in *Firre* KO mice^32,33^. Local cis-effects have also been demonstrated for the imprinted lncRNAs *Airn* and *Kcnq1ot1*, which recruit the PRCs via hnRNPK to silence Mb-sized regions on one allele in cis^39^. Our results based on KD and transgenic rescue reveal dose-dependent effects of *Firre* RNA on H3K27me3 enrichment on the Xi, reminiscent of the dose-dependent silencing effects of *Airn* and *Kcnq1ot1*^39^.

The nucleolus has emerged as a platform for the organization of chromatin enriched in repressive histone modifications^16–18,73^. For example, loss of NPM1 results in deformed nucleoli and redistribution of H3K27me3^74^. Depletion of *Firre* RNA limits association of the Xi to the nucleolus and nuclear periphery, supporting coordinated roles in Xi positioning and maintenance of H3K27me3^20^. Two studies corroborate our findings: indeed, relocation of the Xi to the nucleolus during the cell cycle is required to maintain H3K27me3, and sparser H3K27me3 on the Xi occurs when it is kept away from the nucleolus or nuclear periphery^75,76^. Deletion of *Xist* also decreases both H3K27me3 enrichment and nucleolar association of the Xi^77^. However, we did not observe disruption of *Xist* RNA expression nor coating of the Xi in Δ*Firre*^Xa^ nuclei, suggesting that *Firre* acts independently of *Xist*. On the other hand, CTCF, a protein that facilitates interactions between chromatin and the nucleolus may act in concert with *Firre* to help Xi positioning^51^. In addition to CTCF-DNA interactions CTCF-RNA interactions via specific zinc fingers represent important structural components of genome organization^78^. Such dual interactions characterize *Firre* since CTCF not only binds to the locus on the Xi, but also interacts with the RNA^20,21,62^. Thus, in the absence of *Firre* RNA disrupted Xi anchoring to the nucleolar periphery could result from the significant loss of CTCF binding observed along the entire Xi, including at the *Firre* locus. Supporting this notion, RNA depletion using RNase A disrupts the local chromatin environment around CTCF binding sites and the structural integrity of heterochromatin^38^. We cannot exclude other heterochromatin factors, for example, EZH1 or histone H1, both downregulated in Δ*Firre*^Xa^ and rescued in transgenic lines^79,80^. Considering the 3D structure of the Xi, the *Firre* locus helps insulate the two superdomains of the Xi perhaps via CTCF binding and acts in synergy with *Dxz4* to compact the Xi perhaps via a superloop with *Dxz4*^27^.

Our rescue experiments imply that a subset of *Firre* exons are sufficient for partial maintenance of H3K27me3 on the Xi and its location in the nucleus. It will be interesting to map additional functional units within the *Firre* locus, which may provide a more complete rescue. The *Firre* locus harbors several conserved local repeats and many of these do not overlap exons, except for the RRD repeats that display 65% identity between human and mouse, which makes them good candidates for the rescue function observed^21,23^. Notably, these repeats bind the nuclear matrix protein hnRNPU known to associate with *Xist*, the Xi, and other genomic regions^20,21,24,81–84^.

Our findings of dysregulated genes implicated in cell cycle and development upon loss of *Firre* RNA support a role in cell growth, a common finding for other lncRNAs^85^. Both *Firre* KO mice and mice with *Firre* overexpression have abnormalities specifically in hematopoiesis, implying the importance of *Firre* dosage^32,33^. In human loss of *FIRRE* RNA causes dysregulation of inflammatory gene expression, while amplification of *FIRRE* causes congenital abnormalities and is associated with decreased survival rates in cancer, further supporting *FIRRE* dosage effects^24,85,86^.

Despite ample evidence supporting their biological relevance in cell systems lncRNAs are often dispensable for survival of vertebrate organisms. For example, mice homozygous for deletions of regions harboring 1243 noncoding sequences had no distinguishable phenotypes^87^. Similarly, mice with deletions of nine conserved lncRNAs including *Malat* were viable with no obvious abnormalities^50,88^. *Firre* KO mice have few abnormal phenotypes except for abnormalities in B- and T-cell physiology^33^. This dearth of phenotypes could be explained by alternative pathways, possibly other lncRNAs that may compensate during development, providing redundancy in the system^89^. A recent publication reports that following 10 days of differentiation, mouse ES cells with a *Firre* deletion on the Xa, the Xi or on both alleles show no effects on H3K27me3 on the Xi, which differs from our results based on mutations induced in differentiated fibroblasts^35^. Thus, phenotypic effects apparently depend on the developmental timing of *Firre* mutations, and mutations at early stages may allow compensatory pathways to effectively help survival. Indeed, *Firre* KO ES cells display a marked decrease in growth rate, supporting the existence of critical mechanisms for survival of subsets of cells and subsequent differentiation/development^23^. In addition, differences in the effects of *Firre* mutations may reflect developmental stage-specific composition of PRC complexes^90^.

Another important consideration is cell type specificity. There was no apparent loss of H3K27me3 on the Xi in brain, kidney, and liver from *Firre* KO mice. However, a local disruption of H3K27me3 around *Firre* was found in CLPs and CD8 + T cells, suggesting that *Firre* RNA may act in cis in certain cell types. In a CRISPRi phenotypic screen a majority of lncRNAs only displayed phenotypes in a single cell type^50^. A new approach, PIRCh-seq, designed to find association between RNA and specific chromatin modifications reports *Firre* interactions with H3K27me3 chromatin in MEFs, but not in ES cells, highlighting differences between cell types^91^. Furthermore, mouse and human *Firre*/*FIRRE* exhibit diverse expression patterns across tissues, e.g., enrichment in neural crest but depletion in lung fibroblasts^23^. *Firre* KO mice show organ-dependent gene dysregulation, with a larger number of dysregulated genes in spleen, which can be rescued by ectopic *Firre* expression supporting a trans-acting role in vivo^32,33^. The gene expression changes we observed in kidney-derived Patski cells differ from those reported in *Firre* KO mouse spleen, again suggesting tissue-specificity. In immune cells where *Firre* RNA exerts a local cis-effect on the Xi, it remains to be determined whether *Firre* might be expressed from the Xi. Interestingly, female lymphocytes lack *Xist* clouds and H3K27me3 foci, and show reactivation of the Xi^92^. Further analyses to distinguish alleles is needed to better understand *Firre* local effects in immune cells.

## Methods

### Cell lines

The Patski cell line, originally derived from embryonic kidney (18.5 dpc) from a cross between a female C57BL/6 J (BL6) with an *Hprt*^*BM3*^ mutation and a male *Mus spretus* (*spretus*), was previously selected in HAT (hypoxanthine-aminopterin-thymidine) medium so that the Xi is always from BL6^93,94^. Primary MEF cultures were derived from a 13.5 dpc female embryo resulting from a BL6 × *Mus spretus* cross, which results in skewed inactivation of the *spretus* X chromosome due to an *Xist* mutation on the BL6 X chromosome^95^. Primary MEFs were also derived from BL6 × *Mus spretus* and BL6 × *Mus castaneus* 13.5 dpc female F1 embryos with random XCI. Cells were cultured as described and the presence of normal X chromosomes verified by karyotyping^29^.

For ectopic *Firre* expression assays in Patski cells and in MEFs, a mouse *Firre* cDNA plasmid (mtransgene; Dharmacon BC055934) or a human *FIRRE* cDNA plasmid (htransgene; Dharmacon BC038558) were each transfected together with the selectable marker pPGK-Puro plasmid (gift from R. Jaenisch; Addgene 11349) into Δ*Firre*^Xa^ cells using lipofectamine 3000 (Invitrogen). Blast searches were performed to map homology of the cDNAs to the reference genomes (Supplementary Fig. 1b). After transfection, Δ*Firre*^Xa+mtransgene^ and Δ*Firre*^Xa+htransgene^ were selected in Eagle’s medium with 2 µg/ml puromycin for 72 h, followed by recovery in Eagle’s medium with 1 µg/ml puromycin for 10 days. A Δ*Firre*^Xa+mtransgene^ clone with high *Firre* expression was also isolated.

### KO mouse tissues and isolation of CLPs and CD8 + T cells

Mice used in this study were maintained in pathogen-specific-free facilities under the supervision of either the University of Washington Institutional Animal Care and Use Committee (Protocol number 2254) or Harvard University’s Institutional Animal Care Committee. We complied with the ethical regulations for animal testing and research in accordance with these committees. Tissues collected from a *Firre* KO mouse model included liver, kidney, and brain from heterozygous (*Firre*^+/−^) and homozygous (*Firre*^−/–^) mutants, and from control female mice verified by genotyping^33^. Ectopic expression of *Firre* induced by doxycycline (DOX) injection in mice was done as described^33^. MEFs were derived from mutant (*Firre*^+/−^, *Firre*^−/−^) and control female 13.5 dpc embryos.

We isolated CLPs live [Lin–Sca-1locKitloIL7Rα+] by fluorescence-activated cell sorting (FACS) from WT (age: 35–43 days) and *Firre*^−/−^ (age: 35 days) female mice to generate two replicate samples per genotype, consisting of two mice per replicate. Bone marrow was isolated from both femurs and tibias of each mouse by removing the end caps and flushing the bone marrow with a 27 G syringe containing staining media (DMEM, Gibco, 11995–073) with 5% fetal bovine serum (FBS, Gibco, 26140079) and 10 mM EDTA into a 50 mL conical tube. Cells pelleted by centrifugation at 1200 rpm were subjected to lineage depletion according to the manufacturer’s protocol (Miltenyi Biotec, 130–090–858). Lineage-depleted bone marrow was resuspended with the following antibodies (1:100): PE/Cy7 anti-mouse CD127 (IL-7Rα) clone A7R34 (Biolegend, 135014), Alexa Fluor 488 anti-mouse CD117 (c-Kit) clone 2B8 (Biolegend, 105816), PE/Dazzle-594 anti-mouse Ly-6A/E (Sca-1) clone D7 (Biolegend, 108138), APC anti-mouse CD34 clone HM34 (Biolegend, 128612), PE anti-mouse CD135 clone A2F10 (Biolegend, 135306), and Pacific Blue anti-mouse Lineage Cocktail (20 μL per 1 × 106 cells) clones 17A2/RB6-8C5/RA3-6B2/Ter-119/M1/70 (Biolegend, 133310). Zombie Aqua Fixable Viability Kit (Biolegend, 423101) was used as a viability stain according to the manufacturer’s protocol. Samples were incubated on ice in the dark for 60 min, washed twice with staining media, and resuspended before sorting by FACS (BD Aria).

To isolate CD8 + T-cells peripheral blood was collected from mice by cardiac puncture into a tube containing 4% citrate. Red blood cells were lysed for 15 min at room temperature using BD Pharm Lyse (BD, 555899). Cells were washed twice with staining media and the following antibodies were added (1:100) to each sample prior to incubation for 30 min at room temperature: PE anti-mouse CD3 clone 17A2 (Biolegend, 100205), Alexa Fluor 700 anti-mouse CD8a clone 53–6.7 (Biolegend, 100730), APC anti-mouse CD19 clone 6D5 (Biolegend, 115512), Alexa Fluor 488 anti-mouse NK-1.1 clone PK136 (Biolegend, 108718), PE/Dazzle-594 anti-mouse CD4 clone GK1.5 (Biolegend, 100456), TruStain FcX (anti-mouse CD16/32) clone 93 (1:50) (Biolegend, 101319). Zombie Aqua Fixable Viability Kit (Biolegend, 423101) was used as a viability stain. Cells were washed twice with staining media and sorted by FACS (BD Aria).

### Allele-specific CRISPR/Cas9 editing and RNAi KD

For allele-specific CRISPR/Cas9 editing of the endogenous *Firre* locus, three highly specific sgRNAs with BL6 or *spretus* SNPs at the PAM site and with low off-target scores were chosen and aligned back to the reference genome using BLAT (UCSC) to verify specificity (Supplementary Data 1). The sgRNAs cloned into p × 330 plasmids (Addgene) were transfected into WT Patski cells using Ultracruz reagents (Santa Cruz). A Patski line with a deletion of *Dxz4* was also transfected to generate a double-mutant Δ*Firre*^Xi^/Δ*Dxz4*^Xi^ ^29^. Single-cell derived colonies were selected and deletions or inversions of the targeted *Firre* locus verified using PCR and Sanger sequencing to confirm allele-specific editing (Supplementary Data 2). *Firre* RNAi KD was performed as described^20^. Cells were harvested after double shRNA/siRNA treatment and qRT-PCR performed to verify KD efficiency.

### Immunofluorescence, RNA-FISH, and DNA-FISH

Immunofluorescence was done on cells grown on chamber slides, fixed with paraformaldehyde, permeabilized, and blocked as described previously^20^. Mouse liver, kidney, and brain were embedded in a cassette and sectioned by the University of Washington histopathology service center. Tissue sections (5 µm) were permeabilized using 0.5% Triton X-100 for 10 min and fixed in 4% paraformaldehyde for 10 min. After incubation with a primary antibody specific for H3K27me3 (Upstate/Millipore, #07–449), H2AK119ubi (Cell Signaling, #8240 S), macroH2A (Abcam, #ab37264), or NPM1 (Abcam, #ab10530) overnight at 4 °C in a humidified chamber cells/tissue sections were washed in 1× PBS (phosphate-buffered saline) buffer and incubated with a secondary antibody conjugated to Texas Red (anti-rabbit, Vector, # TI-1000) or fluorescein (anti-mouse, Vector, # FI-2100). RNA-FISH for *Xist* was done using a labeled 10 kb cDNA plasmid (pXho, which contains most of *Xist* exon 1) as described^20^. RNA-FISH for *Firre* was done in a clonal Δ*Firre*^Xa+mtransgene^ line that overexpresses *Firre* RNA using a labeled plasmid probe containing *Firre* cDNA. DNA-FISH was done using labeled BAC probes containing *Firre* (RP24-322N20) or *Dxz4* (RP23-299L1)^29^. Images were acquired with a Zeiss fluorescence microscope equipped with an image capture system ZEN 2.3.

Slides were examined by fluorescence microscopy to score the number of nuclei with enrichment in each histone modification on the Xi, using *Xist* RNA-FISH for Xi identification. A minimum of 300 nuclei were scored per cell type by at least two observers. Measurements of overall H3K27me3 staining intensity outside the Xi cluster were done in a minimum of 300 nuclei using ImageJ^36^. To minimize experimental variance, a mixture of 80% Δ*Firre*^Xa^ and 20% WT cells were grown in the same chamber prior to immunostaining with H3K27me3 and a control histone panH4 (Abcam ab10158), together with DNA counterstaining using Hoechst 33342. Selected nuclear areas away from the Xi were used for measurement of H3K27me3 median intensity, with same-area normalization to either Hoechst 33342 or panH4 staining. Comparisons between WT and Δ*Firre*^Xa^ were done by calculating either the H3K27me3 staining intensity versus Hoechst 33342 and panH4 for WT and Δ*Firre*^Xa^ nuclei separately, or the relative H3K27me3 staining intensity in Δ*Firre*^Xa^ versus WT nuclei present in the same microscope field. The location of the Xi with respect to the nuclear periphery and the edge of the nucleolus (labeled with NPM1) was recorded in at least 300 nuclei.

### Allelic in situ Hi-C, CUT&RUN, ATAC-, RNA-, and ChIP-seq

In situ DNase Hi-C was done on intact nuclei from Δ*Firre*^Xa^, Δ*Firre*^Xi^, Inv*Firre*^Xi^, and WT as described^29,30^. Hi-C libraries were sequenced using 150 bp paired-end reads (Supplementary Data 11). ATAC-seq on Δ*Firre*^Xa^, Δ*Firre*^Xi^, Inv*Firre*^Xi^, Δ*Firre*^Xi^/Δ*Dxz4*^Xi^, and WT, and RNA-seq on Δ*Firre*^Xa^, Δ*Firre*^Xa+mtransgene^, and WT were done as previously described^29^. ChIP-seq was done on Δ*Firre*^Xa^ and WT using an antibody for H3K27me3 and an established protocol^20^. CUT&RUN was done on Δ*Firre*^Xa^, Δ*Firre*^Xa+mtransgene^, and WT using an antibody for CTCF (Upstate/Millipore, 07-729-25UL), and on Δ*Firre*^Xa^ and WT using an antibody for SUZ12 (Abcam ab12073) using a published protocol^96^. H3K27me3 ChIP analyses of CLPs and CD8 + T cells were done using the TrueMicroChIP kit (Diagenode, C01010130) according to the manufacturer’s protocol. Briefly, we sheared fixed chromatin from ~32,000 CLPs and ~50,000 CD8 + T cells in separate 1.5 mL TPX plastic tubes (Diagenode, C3001001050) using the following cycles on a Bioruptor (Diagenode): eight cycles of 5 min (30 ON, 30 OFF). Between each cycle samples were placed on ice. Sheared chromatin was then immunoprecipitated using 1 µL of H3K27me3 antibody (Diagenode, C15410069), retaining 10% of the chromatin as input. ChIP products were eluted in 12 µL and libraries prepared using the MicroPlex Library Preparation kit v2 (Diagenode, C05010012). Ten microleter of ChIP products were used as input, iPCRtagT1-T12 were used for 12 amplification cycles. After library clean up, the libraries were analyzed on a BioAnalyzer DNA-HS chip.

ATAC-seq, ChIP-seq, and CUT&RUN libraries for Patski cells were sequenced as 75 bp pair-end reads (Supplementary Data 11). RNA-seq libraries for Patski cells were sequenced as 75 bp single-end reads (Supplementary Data 11 ). Sequencing datasets were analyzed to assign reads to the *spretus* or BL6 genomes using a previously developed allele-specific data analysis pipeline^29^. RNA-seq reads were mapped to the UCSC mm10 (NCBI build v38) refSeq BL6 mouse transcriptome. Tophat2 (v 2.0.12) (calling bowtie2 (v2.2.3)) was used to perform single-end mapping allowing six mismatches. Mapped reads were assigned to refSeq genes using HT-seq(v0.11.0) and counts were converted into TPMs using custom R scripts. DE analysis was performed using DESeq2. ATAC-seq, ChIP-seq, and CUT&RUN reads were mapped to the BL6 mouse genome using the NCBI build v38/mm10 reference genome assembly obtained from the UCSC Genome Browser using BWA-MEM (v0.7.3) in paired-end mode using default parameters. Peaks were called using MACS2. DNase Hi-C reads were mapped to the BL6 genome using the NCBI build v38/mm10 reference genome assembly obtained from the UCSC Genome Browser and a pseudo-spretus genome using BWA-MEM (v0.7.3) in single-end mode using default parameters^29^. Micro-RNA-seq (less than 200nt) was done in Δ*Firre*^Xa^ and WT Patski cells by BGI Genomics (https://www.bgi.com/us/). ChIP-seq libraries for CLPs and CD8 + T cells were sequenced as 150 bp pair-end reads. Sequencing datasets were analyzed to assign reads to the BL6 genome using the NCBI build v38/mm10 reference genome assembly obtained from the UCSC Genome Browser using Bowtie2. Differential tracks and heatmaps were generated by Deeptools. GO analysis was done using http://geneontology.org/.

## Supplementary information

Supplementary Information

Description of Additional Supplementary Files

Supplementary Data 1–11

## Data Availability

All sequencing data that support the findings of this study have been deposited in the National Centre for Biotechnology Information GEO and are accessible through the GEO SuperSeries “GSE59779”. Publicly available RNA Immunoprecipitation data for Fig. 2i were obtained from NCBI GEO “GSE118402” (RIP-seq_TSC HNRNPK) and “GSE137491” (WT_1_ESC_SUZ12_RIPseq and WT_1_ESC_EZH2_RIPseq). All other data and the scripts used for the analyses that support the findings of this study are available within the article and its Supplementary Information files or from the corresponding authors upon reasonable request. Source data are provided with this paper. A reporting summary for this Article is available as a Supplementary Information file. Source data are provided with this paper.

## Source data

Source Data
