# Supplementary material for: Trans- and cis-acting effects of Firre on epigenetic features of the inactive X chromosome
Source: Nat Commun. 2020 Nov 27;11:6053. doi: 10.1038/s41467-020-19879-3 (PMC7695720; doi:10.1038/s41467-020-19879-3)
Supplement: Supplementary file 1 — Supplementary Information [file 41467_2020_19879_MOESM1_ESM.pdf]

Supplementary Information

Trans- and cis-acting effects of *Firre* on epigenetic features of the inactive X chromosome

Fang, Bonora et al.

Supplementary Figures 1-7

Supplementary References

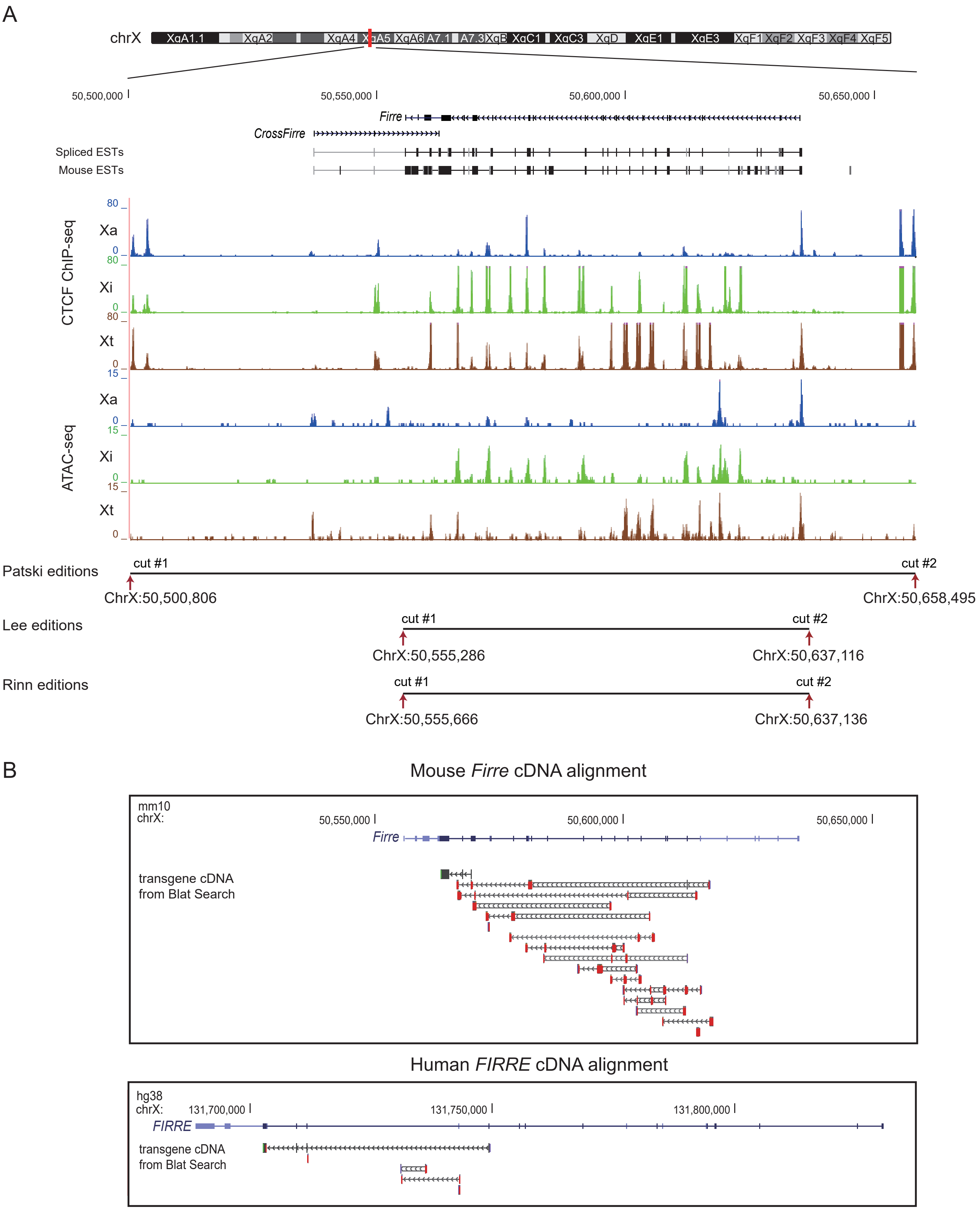

Supplementary Figure 1. Location of existing *Firre* deletions and of mouse *Firre* cDNA and human *FIRRE* cDNA. A. Genome browser map around the *Firre* and *CrossFirre* loci indicating the extent of known deletions compared to the deletion we created33,35. ATAC-seq and ChIP-seq tracks are shown for reference. Note that the *Firre* locus on the Xi exhibits dense CTCF binding and ATAC-seq peaks. Additional CTCF peaks are present on both Xa and Xi at locations ~55kb upstream and ~23kb downstream of *Firre* locus, respectively. **B**. Blast results of alignment of the mouse *Firre* cDNA and of the human *FIRRE* cDNA to the mouse and human reference genomes. The mouse *Firre* cDNA perfectly overlaps a small region at the 3'end of one of the known transcripts. The mouse cDNA also shows homology to multiple sequences distributed over the *Firre* locus, except in the region covering the 5 5' end exons. The human *FIRRE* cDNA overlaps four of the exons at the 3'end of one of known transcripts, but shows no homology to 12 5' end exons.

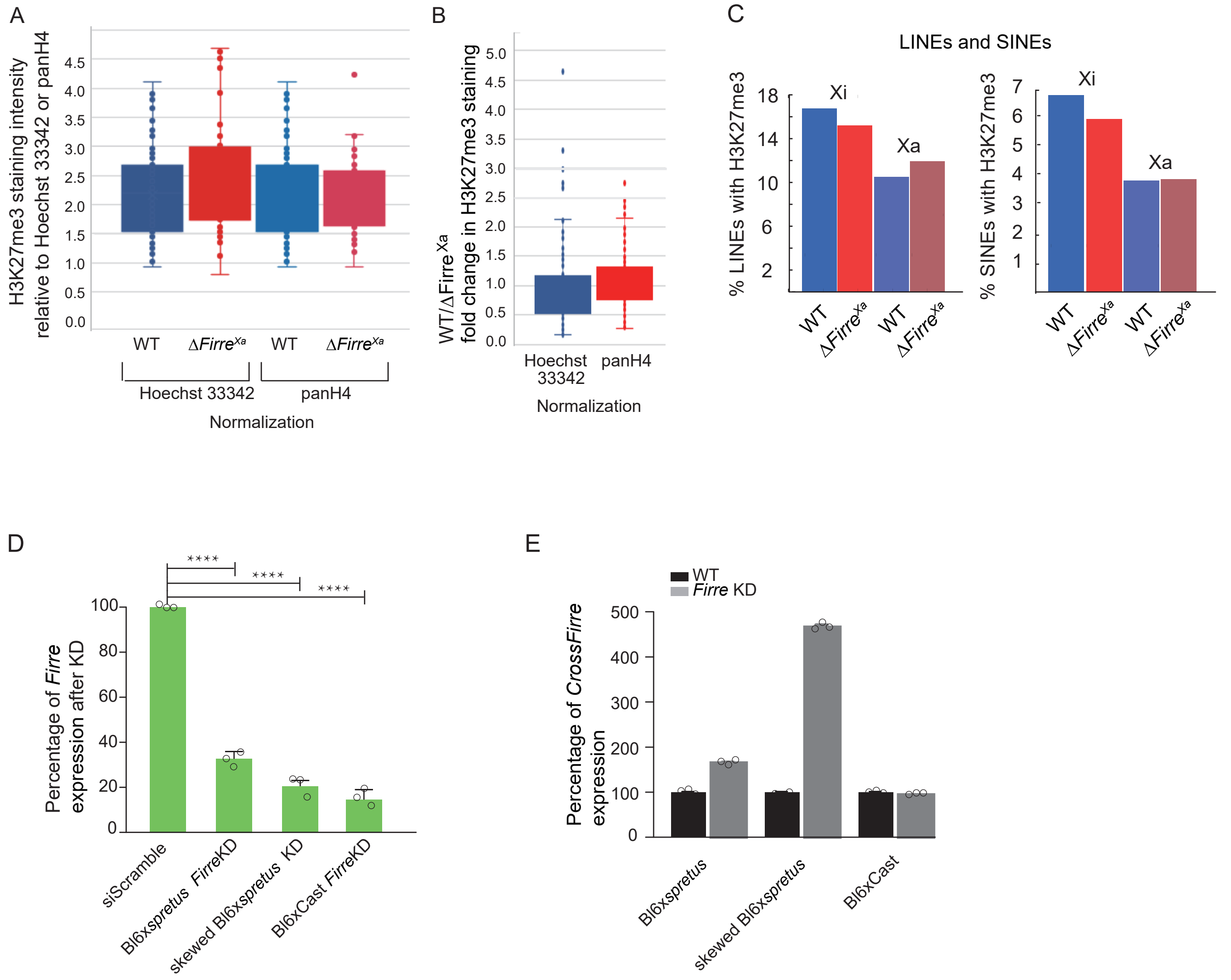

Supplementary Figure 2. A-B. H3K27me3 immunostaining in WT and Δ*Firre*^{Xa} nuclei outside the Xi. C. H3K27me3 ChIP-seq at LINEs and SINEs in WT and ΔFirreXa. D-E. *CrossFirre* and *Firre* expression changes in KD MEFs. A. Box plots of the intensity of H3K27me3 staining relative to Hoechst 33342 or panH4 staining measured by ImageJ for WT (blue) and Δ*Firre*^{Xa}(red). No significant differences were seen (p value=0.8690 for Hoechst normalization; p value=0.9585 for panH4 normalization with two-tail t test). A total of >300 nuclei were scored per cell type over 3 independent experiments. The boxes demarcate the interquartile range (IQR) with median. Whiskers are ±1.5 times the IQR. Outliers plotted as individual points. **B.** Fold change of the intensity of H3K27me3 staining in Δ*Firre*^{Xa} cells relative to WT cells grown on the same slide and observed in the same microscope field. H3K27me3 staining normalized either to Hoechst 33342 of to panH4. No significant deviations from a ratio of 1 were seen (p value=0.6551 for Hoechst normalization; p value=0.5986 for panH4 normalization with two-tail t test). A total of >300 nuclei were scored per cell type over 3 independent experiments. The boxes demarcate the interquartile range (IQR) with median. Whiskers are ±1.5 times the IQR. Outliers plotted as individual points. **C.** Bar plots of H3K27me3 enrichment measured by ChIP-seq at LINE and SINE repeats in WT (Xi blue, Xa purple) and Δ*Firre*^{Xa} (Xi red, Xa pink). **D, E.** Percentage of expression of *Firre* (D) and *CrossFirre* (E) after *Firre* KD in MEFs derived from an F1 embryo (BL6 x *spretus*), with skewed inactivation of the *spretus* X chromosome, and from F1 embryos either (BL6 x *spretus*) or (BL6 x *castaneus*), with random XCI (see also Table1) compared to WT. *CrossFirre* and *Firre* expression was measured by qRT-PCR in n=3 biologically independent samples per cell type. Data are presented as mean values +/- SEM.

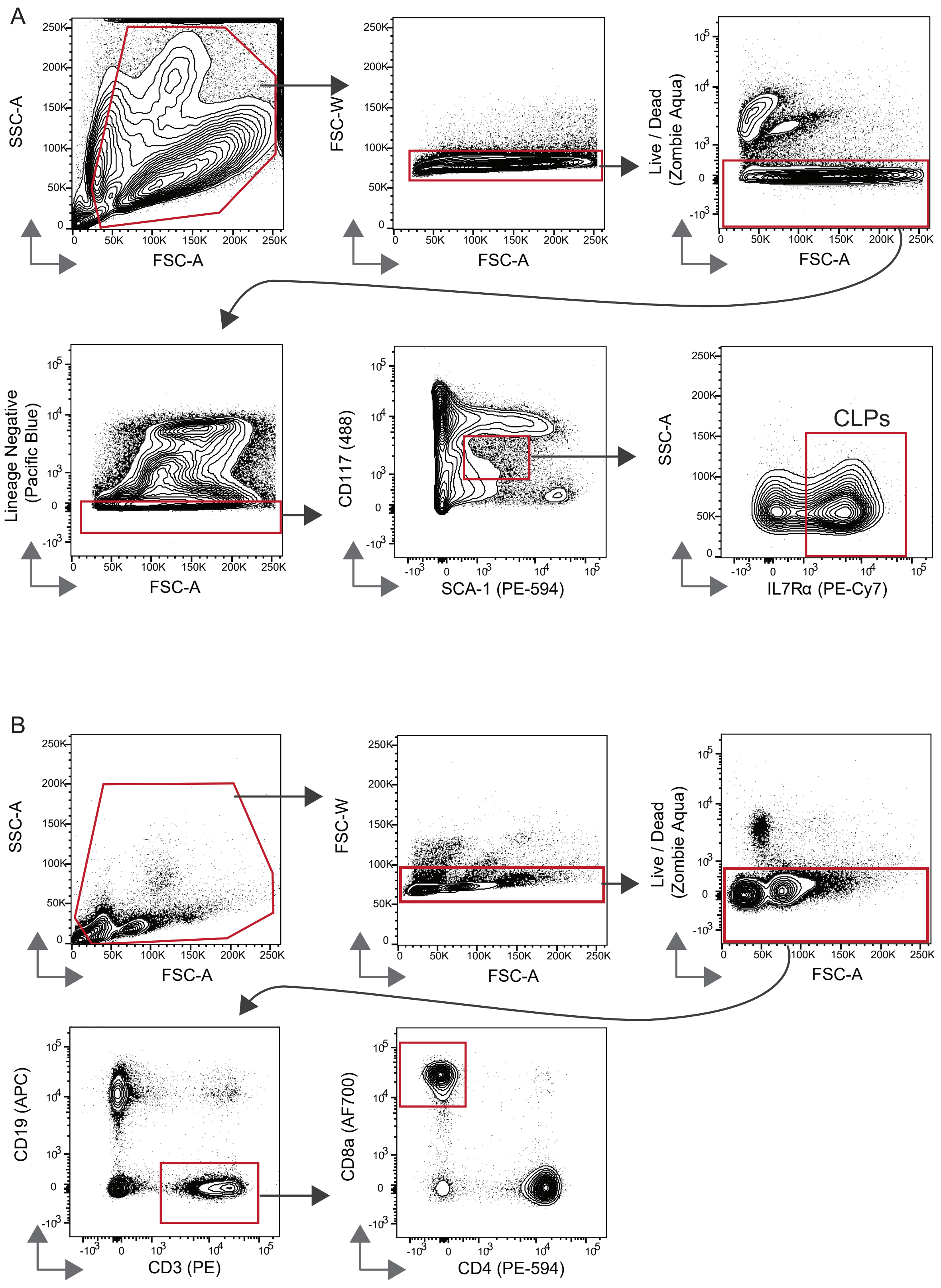

Supplementary Figure 3. Gating strategy for sorting CLPs and CD8+ T-cells. Common lymphoid progenitors (CLPs) **(**6th panel in **A)** and CD8+ T-cells **(**5th panel in **B)** were sorted by fluorescence-activated cell sorting (FACS) prior to H3K27me3 ChIP-seq. Samples shown are from lineage-depleted bone marrow and from peripheral blood in WT mice.

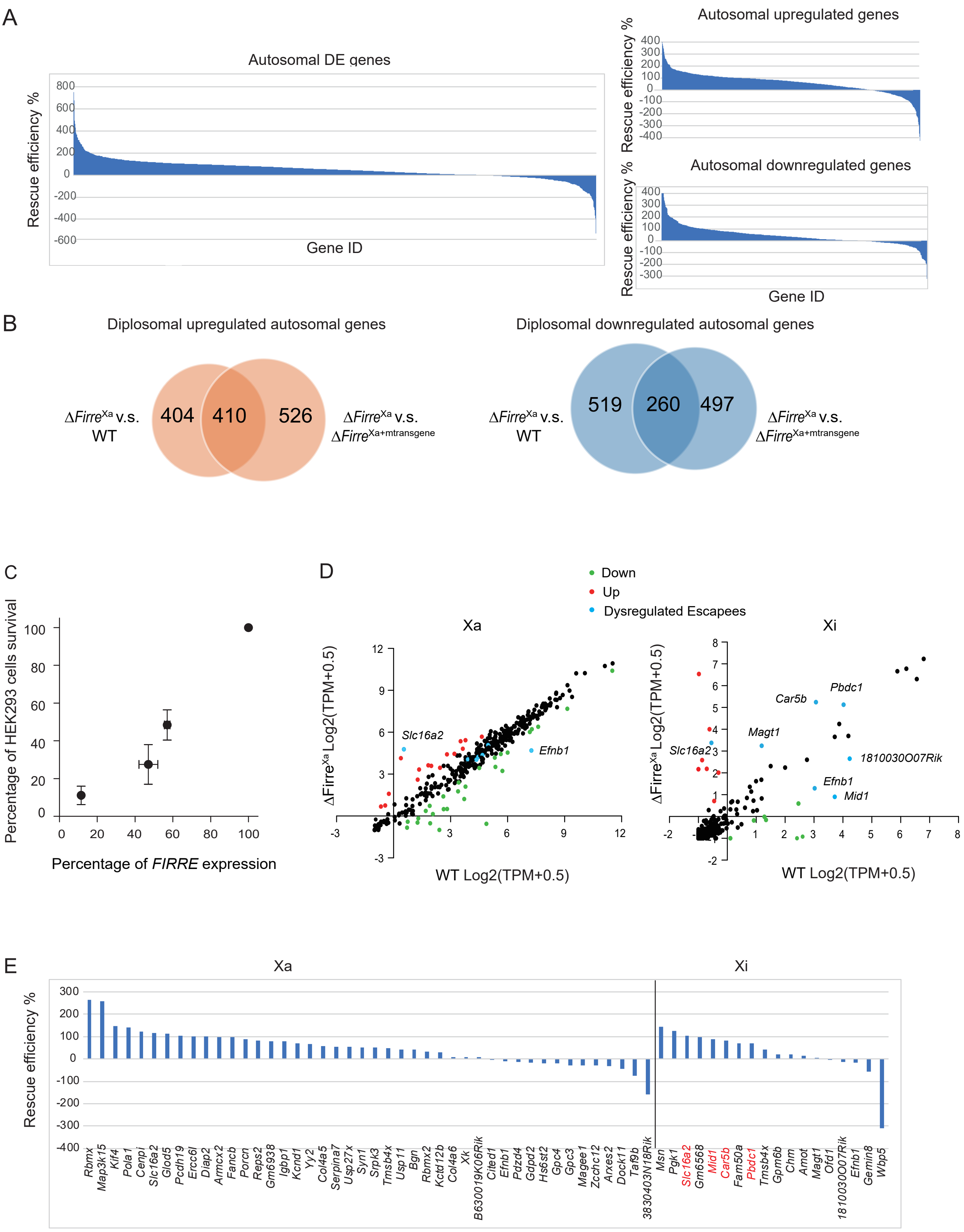
A
Autosomal DE genes
Rescue efficiency %
800
600
400
200
0
-200
-400
-600
Gene ID
Autosomal upregulated genes
Rescue efficiency %
400
300
200
100
0
-100
-200
-300
-400
Autosomal downregulated genes
Rescue efficiency %
400
300
200
100
0
-100
-200
-300
Gene ID
B
Diplosomal upregulated autosomal genes
ΔFirre^Xa v.s. WT
404
410
526
ΔFirre^Xa v.s. ΔFirre^Xa+mtransgene
Diplosomal downregulated autosomal genes
ΔFirre^Xa v.s. WT
519
260
497
ΔFirre^Xa v.s. ΔFirre^Xa+mtransgene
C
Percentage of HEK293 cells survival
100
80
60
40
20
0
0
20
40
60
80
100
Percentage of FIRRE expression
D
Down
Up
Dysregulated Escapees
Xa
ΔFirre^Xa Log2(TPM+0.5)
12
9
6
3
-3
-3
3
6
9
12
Slc16a2
Efnb1
WT Log2(TPM+0.5)
Xi
ΔFirre^Xa Log2(TPM+0.5)
8
7
6
5
4
3
2
1
-2
-2
1
2
3
4
5
6
7
8
Pbdc1
Car5b
Magt1
Slc16a2
1810030O07Rik
Efnb1
Mid1
WT Log2(TPM+0.5)
E
Xa
Xi
Rescue efficiency %
300
200
100
0
-100
-200
-300
-400
Rbmx
Map3k15
Kif4
Pola1
Cenpi
Slc16a2
Glod5
Pcdh19
Ercc6l
Diap2
Armcx2
Fancb
Porcn
Reps2
Gm6938
Igbp1
Kcnd1
Yy2
Col4a5
Serpina7
Usp27x
Syn1
Srpk3
Tmsb4x
Usp11
Bgn
Rbmx2
Kctd12b
Col4a6
Xk
B630019K06Rik
Cited1
Efnb1
Pdzd4
Gdpd2
Hs6st2
Gpc4
Gpc3
Magee1
Zcchc12
Arxes2
Dock11
Taf9b
3830403N18Rik
Msn
Pgk1
Slc16a2
Gm6568
Mid1
Car5b
Fam50a
Pbdc1
Tmsb4x
Gpm6b
Chm
Amot
Magt1
Ofd1
1810030O07Rik
Efnb1
Gemin8
Wbp5

Supplementary Figure 4. **Gene expression changes in Δ*Firre*^{Xa} versus WT are partially rescued by a mouse transgene**. **A**. Rescue efficiency of dysregulated, upregulated, and downregulated autosomal genes in Δ*Firre*^{Xa} cells and WT. 1226 out of 1591 dysregulated genes, 652 out of 813 upregulated genes and 574 out of 778 downregulated genes are rescued or overcorrected (see Supplementary Data 6). **B.** Analysis of dysregulated genes located on autosomes without any aneuploidy in Δ*Firre*^{Xa} cells and WT. The Venn diagrams show the number of upregulated (orange) and downregulated (blue) genes in Δ*Firre*^{Xa} versus WT and in Δ*Firre*^{Xa} versus Δ*Firre*$^{Xa+mtransgene}$. The overlapping gene set represents dysregulated genes in Δ*Firre*^{Xa} that are rescued by transgene expression. **C**. Dose response effect on the percentage of cell survival relative to the remaining percentage of *Firre* RNA after *Firre* KD (shRNA and/or siRNA treatment) in HEK293T cells. *Firre* expression was measured by qRT-PCR and cell survival rate was scored by cell counting in n=3 biologically independent samples per cell type. Data are presented as mean values +/- SEM. **D.** Scatter plots of allelic gene expression from the Xa and Xi for 382 expressed X-linked genes in Δ*Firre*^{Xa} and WT cells. On the Xa 28 and 15 genes are downregulated (green) and upregulated (red) >2-fold, respectively, in Δ*Firre*^{Xa}. On the Xi 10 and 8 genes are upregulated (red) and downregulated (green), respectively, in Δ*Firre*^{Xa}. Dysregulated escape genes are labeled in blue. Allelic TPM (transcripts per million) calculated from RNA-seq based on ratios of Xa/Xi SNP reads. **E**. Rescue efficiency of dysregulated genes on the Xa and on the Xi. 29 out of 43 Xa genes and 12 out of 18 Xi genes are rescued (see Supplementary Data 6). Escape genes are colored in red.

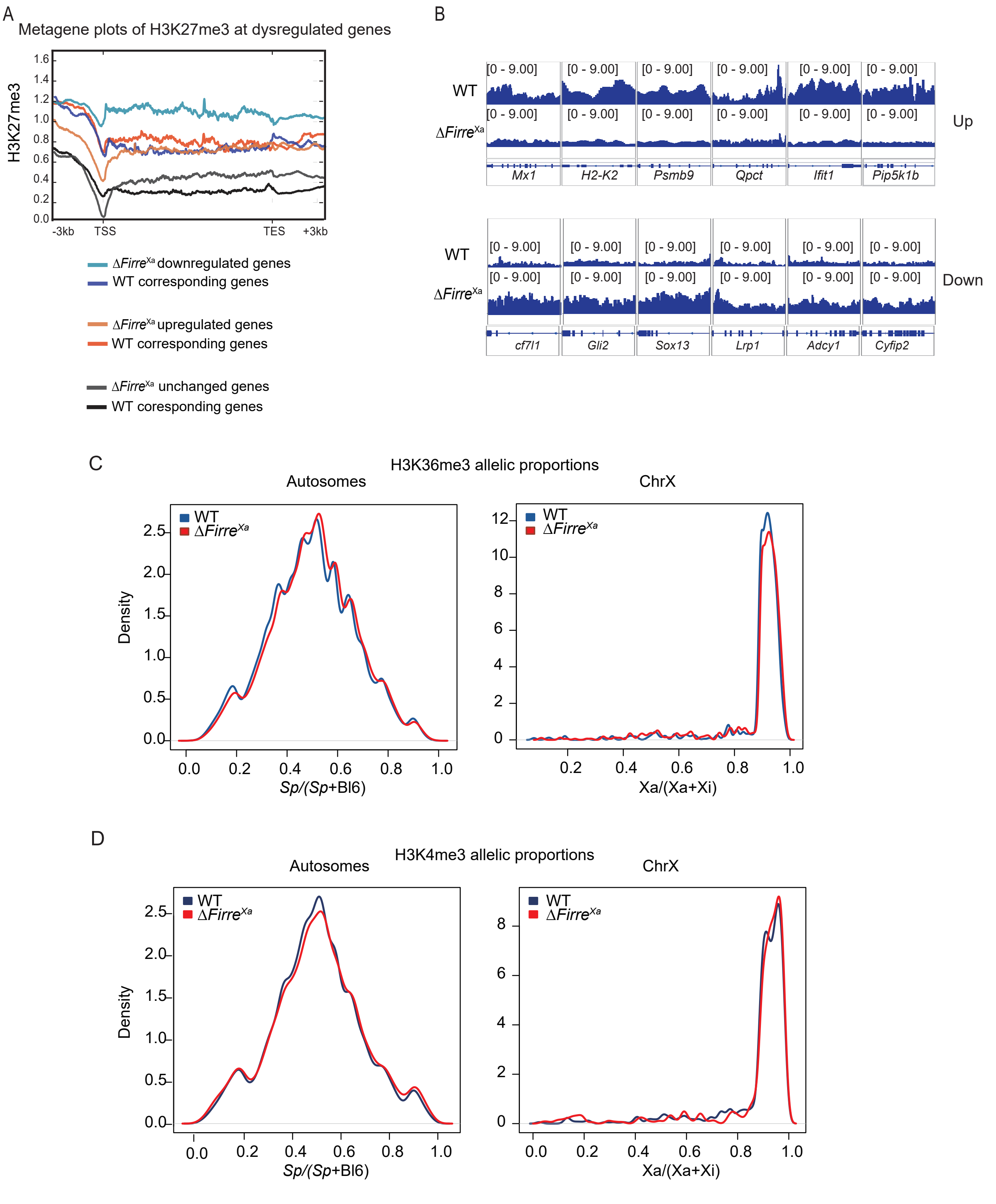

Supplementary Figure 5. H3K27me3 changes at dysregulated genes but no change in H3K36me3 and H3K4me3 enrichment in Δ*Firre*^{Xa}. A. Metagene plots show average H3K27me3 occupancy at genes dysregulated in Δ*Firre*^{Xa} versus WT. Unchanged, downregulated and upregulated genes are color-coded. Average enrichment is shown from the transcription start site (TSS) to the termination site (TES), with 3kb (not at scale) on either side. **B**. Examples of H3K27me3 enrichment changes at six upregulated and six downregulated genes based on ChIP-seq read coverage. **C**. Density histograms of the distribution of allelic proportions of H3K36me3 peaks (spretus/(spretus + BL6)) along the autosomes and the X chromosomes for WT (blue) and ΔFirreXa (red). No significant shift was observed (Wilcoxon test: -log10P = 2.58). **D**. Density histograms of the distribution of allelic proportions of H3K4me3 peaks (spretus/(spretus + BL6)) along the autosomes and the X chromosomes for WT (blue) and Δ*Firre*^{Xa} (red). No significant shift was observed (Wilcoxon tests: -log10P = 0.7).

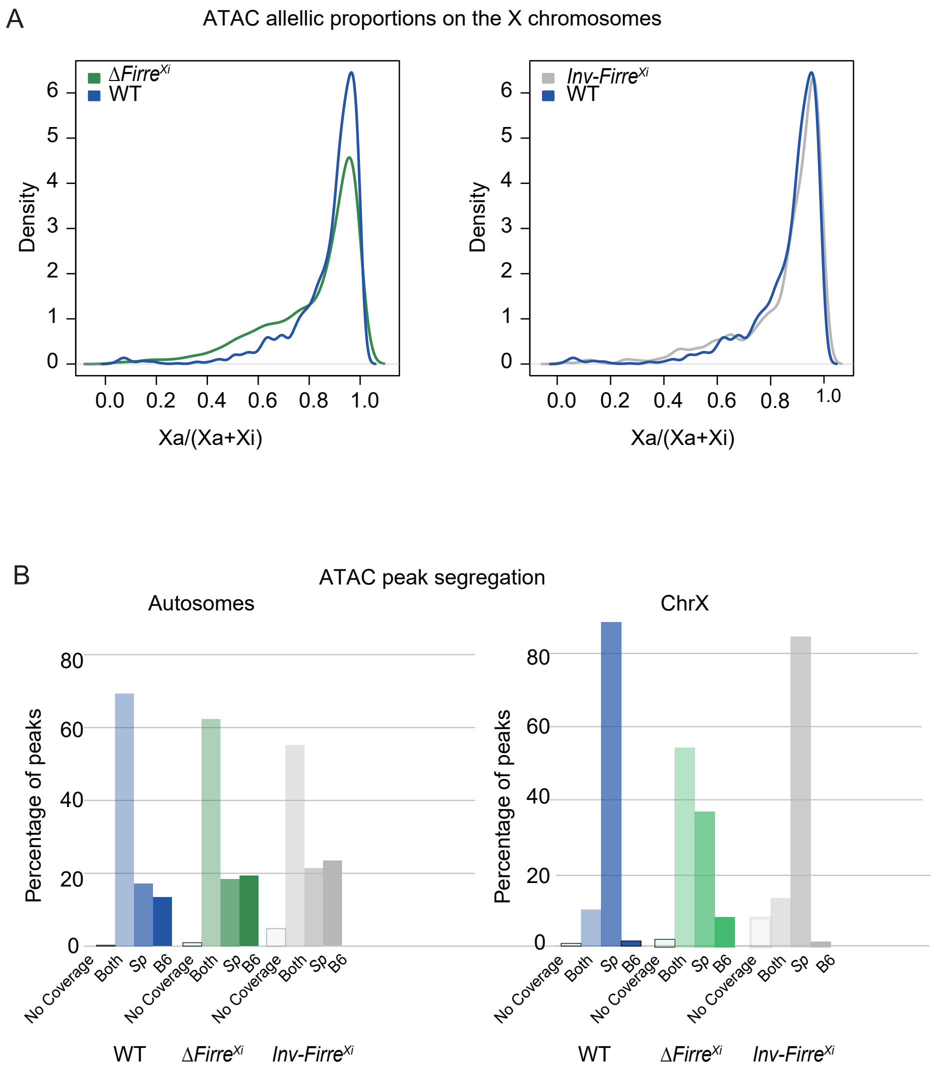

Supplementary Figure 6. No change in chromatin accessibility after *Firre* deletion or inversion on the Xi. **A.** Density histograms of the distribution of allelic proportions (*spretus* /(*spretus* + BL6)) of ATAC peaks along the X chromosomes for WT (blue), Δ*Firre*^{Xi} (green) and Inv*Firre*^{Xi} (grey). No shift is observed (Wilcoxon test: -log10P = 7 for Δ*Firre*^{Xi} and -log10P = 1 for Inv*Firre*^{Xi}). **B.** Percentages of ATAC peaks in WT (blue), Δ*Firre*^{Xi}(green) and Inv*Firre*^{Xi}(grey) along the autosomes and the X chrom-osomes classified as *spretus*-specific, BL6-specific, or at both.

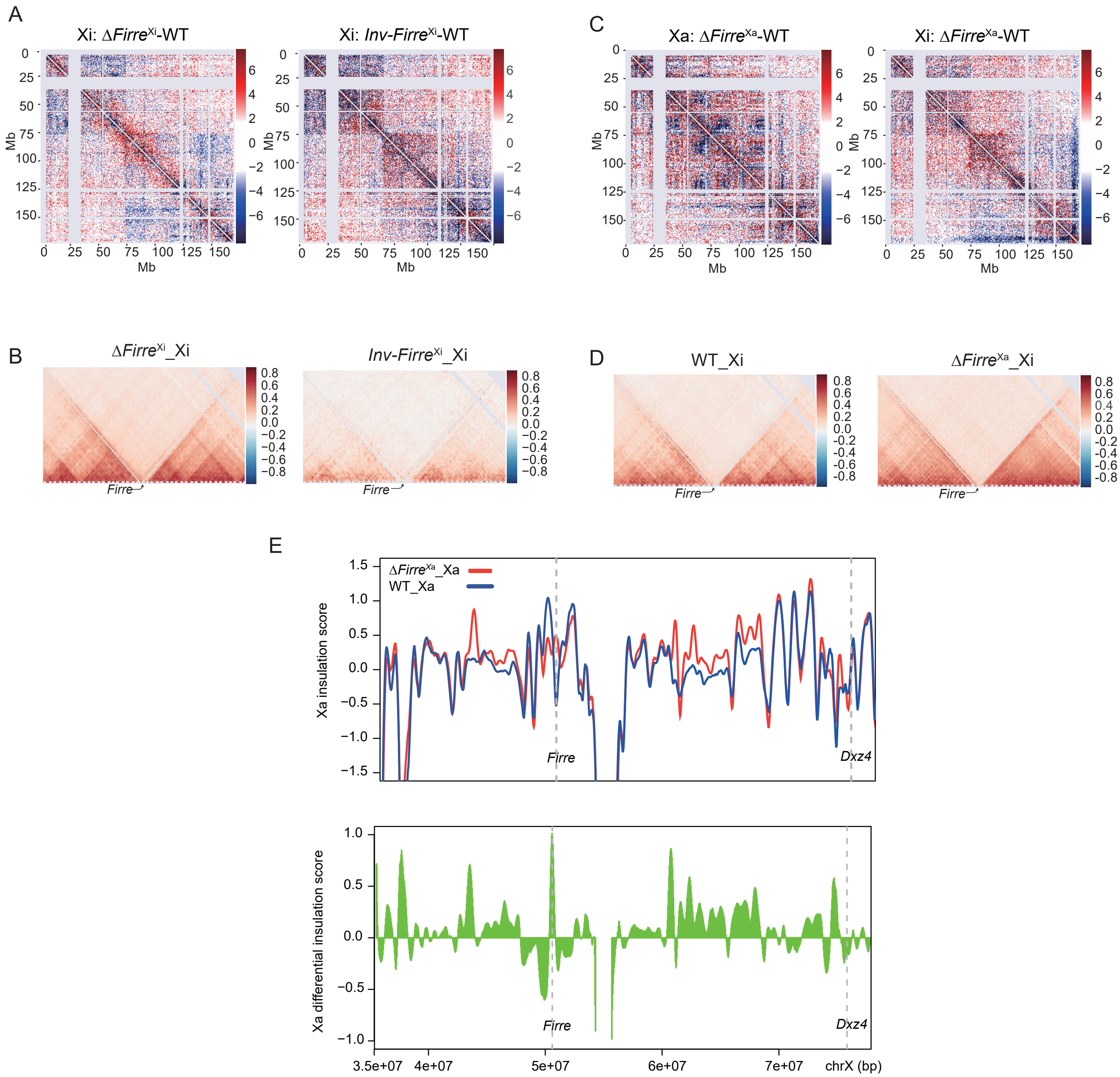

Supplementary Figure 7. Hi-C analyses of WT, Δ*Firre*^{Xa}, Δ*Firre*^{Xi} and Inv*Firre*^{Xi} cell lines. A. Differential contact maps of the Xi at 500kb resolution to highlight differences between Δ*Firre*^{Xi}, Inv*Firre*^{Xi} and WT. Loss and gain of contacts in the ΔFirreXi, Inv*Firre*^{Xi} versus WT appear blue and red, respectively. The color scale shows differential values. **B**. Pearson correlated-transformed contact maps (40kb resolution) for 4Mb around the *Firre* locus highlight persistence of the strong boundary between TADs on the Xi in Δ*Firre*^{Xi} and Inv*Firre*^{Xi}. **C**. Differential contact maps of the Xa and the Xi at 500kb resolution to highlight differences between Δ*Firre*^{Xa} and WT. Loss and gain of contacts in the Δ*Firre*X^{a} versus WT appear blue and red, respectively. The color scale shows differential values. **D.** Pearson correlated-transformed contact maps (40kb resolution) for 4Mb around the *Firre* locus highlight persistence of the strong boundary between TADs on the Xi in Δ*Firre*^{Xa} and WT. E. Insulation score profiles at 40kb resolution for the whole Xa in WT (blue) and Δ*Firre*^{Xa} (red). A differential insulation score profile of the Xa (green) in Δ*Firre*^{Xa} relative to WT based on 40kb resolution is shown below. The position of *Firre* and *Dxz4* is indicated.

Supplementary References

1 Schuettengruber, B., Bourbon, H.M., Di Croce, L. & Cavalli, G. Genome Regulation by Polycomb and Trithorax: 70 Years and Counting. Cell 171, 34-57 (2017).

2 Yu, J.R., Lee, C.H., Oksuz, O., Stafford, J.M. & Reinberg, D. PRC2 is high maintenance. Genes Dev 33, 903-935 (2019).

3 Chittock, E.C., Latwiel, S., Miller, T.C. & Müller, C.W. Molecular architecture of polycomb repressive complexes. Biochem Soc Trans 45, 193-205 (2017).

4 Brockdorff, N. Polycomb complexes in X chromosome inactivation. Philos Trans R Soc Lond B Biol Sci 372(1733):20180021 (2017).

5 Lu, Z., Carter, A.C. & Chang, H.Y. Mechanistic insights in X-chromosome inactivation. Philos Trans R Soc Lond B Biol Sci 372(1733):20160356 (2017)

6 Brockdorff, N., Bowness, J.S. & Wei, G. Progress toward understanding chromosome silencing by Xist RNA. Genes Dev 34, 733-744 (2020).

7 Monfort, A. & Wutz, A. The B-side of Xist. F1000Res 9 F1000 Faculty Rev-55 (2020).

8 da Rocha, S.T. et al. Jarid2 Is Implicated in the Initial Xist-Induced Targeting of PRC2 to the Inactive X Chromosome. Mol Cell 53, 301-16 (2014).

9 Almeida, M. et al. PCGF3/5-PRC1 initiates Polycomb recruitment in X chromosome inactivation. Science 356, 1081-1084 (2017).

10 Geuens, T., Bouhy, D. & Timmerman, V. The hnRNP family: insights into their role in health and disease. Hum Genet 135, 851-67 (2016).

11 Pintacuda, G. et al. hnRNPK Recruits PCGF3/5-PRC1 to the Xist RNA B-Repeat to Establish Polycomb-Mediated Chromosomal Silencing. Mol Cell 68, 955-969.e10 (2017).

12 Pullirsch, D. et al. The Trithorax group protein Ash2l and Saf-A are recruited to the inactive X chromosome at the onset of stable X inactivation. Development 137, 935-43 (2010).

13 Hasegawa, Y., Brockdorff, N., Kawano, S., Tsutui, K. & Nakagawa, S. The matrix protein hnRNP U is required for chromosomal localization of Xist RNA. Dev Cell 19, 469-76 (2010)

14 Hacisuleyman, E. et al. Topological organization of multichromosomal regions by the long intergenic noncoding RNA Firre. Nat Struct Mol Biol 21, 198-206 (2014)

15 Braccioli, L. & de Wit, E. CTCF: a Swiss-army knife for genome organization and transcription regulation. Essays Biochem 63, 157-165 (2019).

16 Xu, N., Donohoe, M.E., Silva, S.S. & Lee, J.T. Evidence that homologous X-chromosome pairing requires transcription and Ctcf protein. Nat Genet 39, 1390-6 (2007)

17 Filippova, G.N. et al. Boundaries between chromosomal domains of X inactivation and escape bind CTCF and lack CpG methylation during early development. Dev Cell 8, 31-42 (2005).

18 Chadwick, B.P. DXZ4 chromatin adopts an opposing conformation to that of the surrounding chromosome and acquires a novel inactive X-specific role involving CTCF and antisense transcripts. Genome Res 18, 1259-69 (2008).

19 Yang, F. et al. The lncRNA Firre anchors the inactive X chromosome to the nucleolus by binding CTCF and maintains H3K27me3 methylation. Genome Biol 16, 52 (2015).

20 Bonora, G. et al. Orientation-dependent Dxz4 contacts shape the 3D structure of the inactive X chromosome. Nat Commun 9, 1445 (2018).

21 Attwooll, C. et al. A novel repressive E2F6 complex containing the polycomb group protein, EPC1, that interacts with EZH2 in a proliferation-specific manner. J Biol Chem 280, 1199-208 (2005)

22 Sánchez, C. et al. Proteomics analysis of Ring1B/Rnf2 interactors identifies a novel complex with the Fbxl10/Jhdm1B histone demethylase and the Bcl6 interacting corepressor. Mol Cell Proteomics 6, 820-34 (2007).

23 Wei, C. et al. RBFox2 Binds Nascent RNA to Globally Regulate Polycomb Complex 2 Targeting in Mammalian Genomes. Mol Cell 62, 982 (2016)

24 Yeo, G.W. et al. An RNA code for the FOX2 splicing regulator revealed by mapping RNA-protein interactions in stem cells. Nat Struct Mol Biol 16, 130-7 (2009).

25 Smola, M.J. et al. SHAPE reveals transcript-wide interactions, complex structural domains, and protein interactions across the Xist lncRNA in living cells. Proc Natl Acad Sci U S A 113, 10322-7 (2016).
26 Kouzarides, T. Chromatin modifications and their function. Cell 128, 693-705 (2007).
