# Supplementary material for: Trans- and cis-acting effects of Firre on epigenetic features of the inactive X chromosome
Source: Nat Commun. 2020 Nov 27;11:6053. doi: 10.1038/s41467-020-19879-3 (PMC7695720; doi:10.1038/s41467-020-19879-3)
Supplement: Supplementary file 2 — Description of Additional Supplementary Files [file 41467_2020_19879_MOESM2_ESM.pdf]

Description of Additional Supplementary Files

Supplementary Data 1. List of sgRNAs for CRISPR/Cas9 editing, and siRNA/shRNAs for KD

Supplementary Data 2. PCR primers

Supplementary Data 3: X-linked genes dysregulated in ΔFirreXa compared to WT

Supplementary Data 4. Predicted novel miRNAs in ΔFirreXa cells

Supplementary Data 5. Summary of proteins associated with PRC that interact with mouse Firre RNA

Supplementary Data 6. List of genes located within ChrX: 36Mb-62Mb

Supplementary Data 7. Categories of genes with expressio n changes in ΔFirreXa, and rescue efficiency using a mouse transgene in ΔFirreXa

Supplementary Data 8. Autosomal genes dysregulated in ΔFirreXa compared to WT

Supplementary Data 9. Autosomal genes on chromosomes without aneuploidy dysregulated in ΔFirreXa cells compared to WT

Supplementary Data 10. GO terms for dysregulated genes in ΔFirreXa as compared to WT, and rescued in ΔFirreXa+mtransgene

Supplementary Data 11. Summary of read counts for in situ DNase Hi-C, RNA-seq, ATAC-seq, H3K27me3 ChIP-seq, CTCF CUT&RUN and SUZ12 CUT&RUN read counts
